# Human EGFRvIII chimeric antigen receptor T cells demonstrate favorable safety profile and curative responses in orthotopic glioblastoma

**DOI:** 10.1002/cti2.1440

**Published:** 2023-03-05

**Authors:** Rebecca C Abbott, Melinda Iliopoulos, Katherine A Watson, Valeria Arcucci, Margareta Go, Hannah E Hughes‐Parry, Pete Smith, Melissa J Call, Ryan S Cross, Misty R Jenkins

**Affiliations:** ^1^ Immunology Division The Walter and Eliza Hall Institute of Medical Research Parkville VIC Australia; ^2^ The Department of Medical Biology University of Melbourne Parkville VIC Australia; ^3^ Structural Biology Division The Walter and Eliza Hall Institute of Medical Research Parkville VIC Australia; ^4^ Myrio Therapeutics Blackburn North, Melbourne VIC Australia; ^5^ Department of Biochemistry and Chemistry Institute for Molecular Science, La Trobe University Bundoora VIC Australia

**Keywords:** CAR T cells, chimeric antigen receptor, EGFRvIII, glioblastoma, immunotherapy

## Abstract

**Objectives:**

Glioblastoma is a highly aggressive and fatal brain malignancy, and effective targeted therapies are required. The combination of standard treatments including surgery, chemotherapy and radiotherapy is not curative. Chimeric antigen receptor (CAR) T cells are known to cross the blood–brain barrier, mediating antitumor responses. A tumor‐expressed deletion mutant of the epidermal growth factor receptor (EGFRvIII) is a robust CAR T cell target in glioblastoma. Here, we show our *de novo* generated, high‐affinity EGFRvIII‐specific CAR; GCT02, demonstrating curative efficacy in human orthotopic glioblastoma models.

**Methods:**

The GCT02 binding epitope was predicted using Deep Mutational Scanning (DMS). GCT02 CAR T cell cytotoxicity was investigated in three glioblastoma models *in vitro* using the IncuCyte platform, and cytokine secretion with a cytometric bead array. GCT02 *in vivo* functionality was demonstrated in two NSG orthotopic glioblastoma models. The specificity profile was generated by measuring T cell degranulation in response to coculture with primary human healthy cells.

**Results:**

The GCT02 binding location was predicted to be located at a shared region of EGFR and EGFRvIII; however, the *in vitro* functionality remained exquisitely EGFRvIII specific. A single CAR T cell infusion generated curative responses in two orthotopic models of human glioblastoma in NSG mice. The safety analysis further validated the specificity of GCT02 for mutant‐expressing cells.

**Conclusion:**

This study demonstrates the preclinical functionality of a highly specific CAR targeting EGFRvIII on human cells. This CAR could be an effective treatment for glioblastoma and warrants future clinical investigation.

## Graphical abstract

GCT02‐EGFRvIII‐specific human CAR T cells demonstrate safe and specific targeting of glioblastoma. The high‐affinity GCT02 CAR T cells engraft, infiltrate the brain tumor and mediate curative responses in two orthotopic glioblastoma models. This study provides the preclinical data package and launchpad for a human clinical trial.

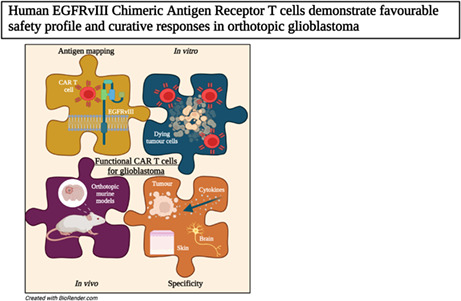

## Introduction

Glioblastoma (GBM) is the most aggressive form of adult brain cancer. The tumor type accounts for nearly half of all adult brain and central nervous system (CNS) tumors,^1^ yet the 5‐year relative survival rate is only 5%.^2^ The current standard of care, the Stupp Protocol, was established in 2005 and added Temozolomide, an oral chemotherapeutic, to surgical resection and locoregional radiotherapy. Whilst providing patients with a small but significant increase in survival compared with radiotherapy alone,^3^ this regimen remains unlikely to be curative, as the diffuse nature of glioblastoma often results in tumor progression.

The above factors highlight the critical need for new glioblastoma therapies to be developed, although one of the challenges to drug development is the unique biology of the brain. The blood–brain barrier (BBB) is the network of vessels which surround, supply and protect the CNS. These vessels greatly restrict the movement of molecules into the CNS, and consequently, can impede the entry of systemically delivered therapeutics into the brain.^4^ Whilst large tumor burdens can perturb normal brain and vascular structure, resulting in leaking of the BBB, the diffuse nature of the tumors results in barrier leakiness in some areas of the brain but not all.^5^, ^6^ Consequently, newly designed therapies should avoid reliance on barrier modification to allow access, but rather develop therapeutics capable of crossing an intact barrier.

Historically, the brain was considered an immune‐privileged site, devoid of immune cells; however, a revised perspective has uncovered a rich lymphatic system. In glioblastoma, four infiltrative immune signatures have been identified,^7^ and immune profiling has revealed elevated CD3^+^/CD8^+^ cells are associated with prolonged survival in GBM patients.^8^ Additionally, several immunosuppressive cell types and secreted factors have been identified in the tumor microenvironment (TME).^8^ These studies highlight the critical role of the immune system in glioblastoma, and with this evidence, it is unsurprising that the number of therapies targeting and enhancing the immune response to glioblastoma has greatly expanded in the past decade.

Genetic engineering of a patient's T cells with a synthetic Chimeric Antigen Receptor (CAR) has gained clinical traction in recent years. The CAR contains an antigen binding domain, which directs the antitumor functions of the T cell towards a malignant cell expressing a selected antigen. Whilst a handful of protein targets have been explored for CAR T cell therapy for GBM,^9^, ^10^, ^11^ a truncation mutation of the epidermal growth factor receptor (EGFR), EGFRvIII, has been of great clinical focus because of its tumor‐restricted expression.^12^ EGFRvIII is expressed in ~ 30% of glioblastoma tumors.^13^ Two EGFRvIII‐specific CAR T cell clinical trials have been conducted, each differing in CAR design. The first product utilised the humanised 3C10 antigen binding domain (humanized clone 2173) and was developed by the University of Pennsylvania and Novartis.^14^ The second CAR contains the 139 binding domain and was developed by the National Cancer Institute.^15^ Whilst these CARs were both shown to be generally safe (dose‐limiting toxicity observed in the 139 trial), neither trial reported objective responses in secondary measures.^16^, ^17^

In addition to antitumor efficacy, product safety measured by inflammatory cytokine release and targeting of healthy tissue are commonly evaluated in CAR T cell development. The brain is a delicate environment, and sustained inflammation triggered by excessive inflammatory cytokine release must be avoided to protect patients from neurological damage; these were therefore important factors to assess in our study.

We recently published a *de novo* generated, high‐affinity EGFRvIII‐specific antigen binding domain; GCT02, and its function as a CAR on murine T cells.^18^ This domain was found to be ~ 300‐fold greater affinity than the reported affinity of the 2173 scFv.^14^ In this study, we found our GCT02 CD8^+^ CAR T cells secreted significantly less quantities of TNF‐α and MIP1α than the 2173 CAR whilst maintaining the capacity to completely clear intracranial glioblastoma tumors in an orthotopic model in NSG mice.^18^ We now demonstrate the function of this construct in primary human T cells. Human GCT02 CAR T cells demonstrated comparable levels of cytotoxicity whilst a reduction in the secretion of undesirable pro‐inflammatory cytokines (compared with the 2173 CAR). We successfully cured mice with established orthotopic EGFRvIII^+^ intracranial tumors using two models. Finally, we mapped the predicted GCT02 binding sites and demonstrated a favorable specificity profile, with no reactivity to primary human keratinocytes known to express high levels of EGFR or healthy astrocytes.

## Results

### The GCT02 binding domain is predicted to be shared between EGFR and EGFRvIII

Previously, a fully human germline scFv library was screened in the Retained Display (ReD) platform for binding to the extracellular domain of recombinant EGFRvIII.^18^ The selected binder was cross‐screened against recombinant EGFR and the resulting binder; GCT02 was characterised for selectivity to EGFRvIII and function as a CAR on murine T cells.^18^ We observed no reactivity of the GCT02 murine CAR T cells to murine cell lines, which did not express the EGFRvIII mutation.^18^ Whilst the CAR demonstrated functional specificity, the binding epitope was unknown. Deep mutational scanning (DMS) was employed to predict the critical binding residues for GCT02. The GCT02 and Cetuximab (commercial anti‐EGFR monoclonal, control with known binding epitope) binders were screened against a retroviral‐based barcoded DMS library of EGFRvIII variants in which single‐site substitutions encompassing all possible mutations were included at positions 1–43 and solvent‐exposed positions from 44 to 348 of the EGFRvIII extracellular domain (Supplementary figure 1).

BW5147 cells expressing the EGFRvIII variants were stained with Cetuximab and GCT02. We chose Cetuximab to report on EGFRvIII surface levels as its binding epitope is known, and single‐site substitutions^19^ have only modest impacts on binding. Cells were sorted based on binding or nonbinding to the antibodies. The frequency of all EGFRvIII variants in each population was determined by counting barcodes present on EGFRvIII mRNA by Illumina sequencing.

**Figure 1:**
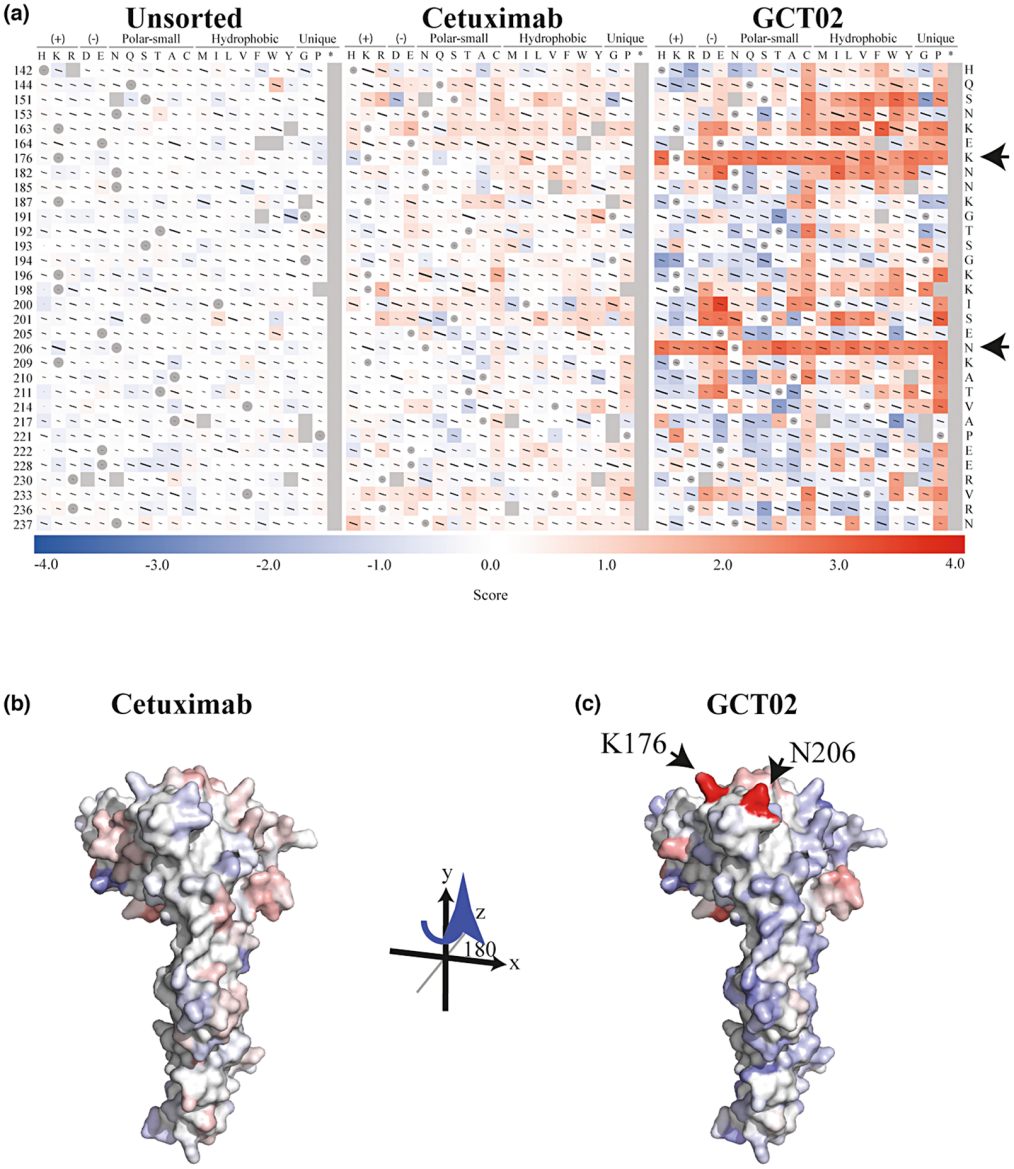
Predicted binding site of GCT02 as determined by Deep Mutational Scanning (DMS). The BW5147 cell line was transduced with the epitope mapping library, labelled with either Cetuximab or GCT02 and sorted for binding or non‐binding. **(a)** Sequence‐Function Maps of unsorted cells compared by Plasmid DNA, Cetuximab stained cells (nonbinding versus binding) and GCT02 stained (non‐binding versus binding) cells. Variant frequency in each cell population was used to calculate Log‐Ratio scores that are used to colour the sequence‐function maps. A score of 1 indicates an approximate 10‐fold enrichment of that variant in the nonbinding population. Positive enrichment scores are coloured red (nonbinding variants), while negative enrichment scores are coloured blue (binding variants). A subset of the 150 positions targeted by DMS are shown here. The full sequence‐function maps can be found in Supplementary figure 2. Lines within each square represent standard error bars, with smaller bars indicating higher confidence. Squares containing a circle show the wild‐type sequence. Grey squares denote no data. Alphafold model of EGFRvIII protein coloured by binding scores of **(b)** Cetuximab and **(c)** GCT02. The Log Ratio enrichment scores of Alanine, Serine, Threonine, Asparagine, Glutamine, Aspartic Acid, Glutamic Acid, Lysine, Arginine and Histidine variants were aggregated by position. The sum of Log Ratio scores was then used to replace the Cα B‐factor of each position in an AlphaFold model of EGFRvIII. Residues that were not targeted in the DMS screen were set to 0. The surface was coloured by B‐factor on a blue‐white‐red spectrum and scaled such that blue and red extended equally into the negative and positive scale and set to maximise the contrast of each dataset: Cetuximab ± 12.69 and GCT02 ± 22.81. Red indicates variants that are enriched in the non‐binding population. Blue indicates variants that are enriched in the binding population. The experiment was performed with three independent BW5147 libraries, each in triplicate.

To ensure that retroviral transduction of the library and puromycin selection before cell sorting did not result in systematic changes in variant frequency, we compared the frequency of variants in the original plasmid stocks used to make retrovirus with the frequency of variants in the mRNA of unsorted cells using the software program Enrich2^20^ (Figure 1a, unsorted). As expected, the sequence‐function map of Plasmid versus unsorted cells showed very little changes in variant frequency. These results indicate that changes in variant frequency among sorted cells will be dependent on antibody staining.

Next, we compared variant enrichment in low versus high cells stained with either Cetuximab or GCT02 (Figure 1a). Variants which lost the ability to bind antibodies will contain mutations that either affect antibody binding directly or cause more global defects in EGFRvIII folding or trafficking to the cell surface. For the latter category of mutation, we would expect that these mutations will be found in the low‐staining population regardless of which antibody is used for staining. Whilst enrichment of GCT02 samples showed higher contrast when viewed on a sequence‐function map, both Cetuximab and GCT02 were sensitive to similar mutations, providing confidence that selection had occurred and that positions of shared enrichment between GCT02 and Cetuximab were likely to cause folding defects. To predict the binding epitope of GCT02, we focussed on enriched positions in the negative population of GCT02 antibody, but not in Cetuximab (Figure 1a, arrows, Supplementary figure 2a). By this criterion, we found two positions in which multiple mutations caused strong enrichment in the low GCT02‐stained population, but not in the Cetuximab‐stained population.

To understand how these residues were clustered on the surface of EGFRvIII, we aggregated Enrich2 scores from each position. To minimise the impact of globally disruptive mutations, we aggregated Alanine, polar and charged mutation scores, which are more likely to be tolerated in surface‐exposed positions. The aggregated scores were used to colour the surface of an AlphaFold^21^ model of the EGFRvIII extracellular domain (Figure 1b and c, Supplementary figure 2b). The most strongly coloured enriched residues clustered on that same face of the Leucine‐rich L2 domain that binds EGF and are close enough to be simultaneously engaged upon antibody binding. From these data, we conclude that GCT02 contacts K176 and N206 (Figure 1a and c, Supplementary figure 2). As these residues are shared between EGFR and EGFRvIII, we cannot definitively rule out some binding to both proteins; however, protein sequence alignment between murine and human EGFR indicates that the GCT02 domain shows limited cross‐reactivity to murine EGFR and is specific to the human protein.

**Figure 2:**
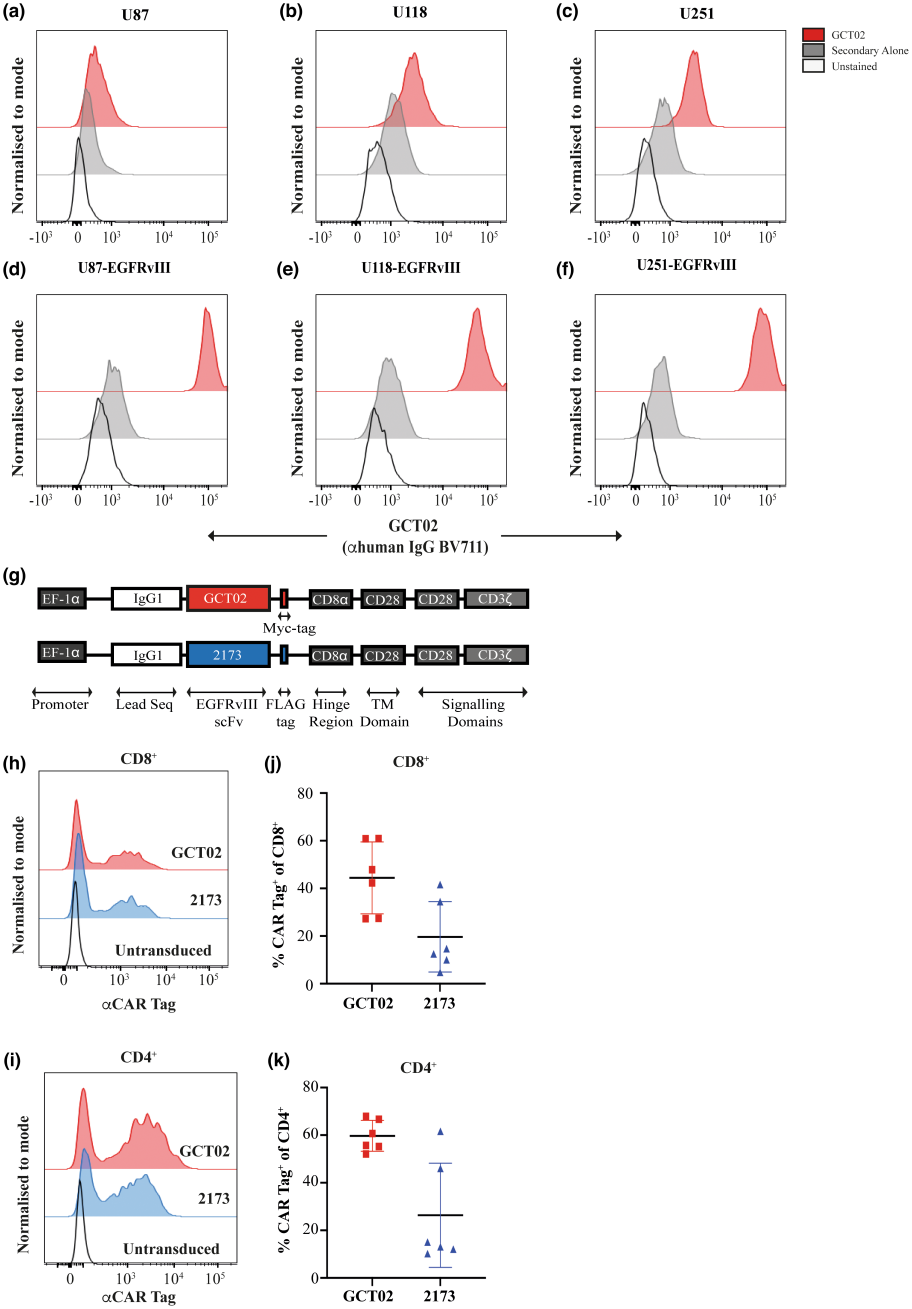
GCT02 binding domain can detect EGFRvIII expression, and the CAR successfully expressed on primary human T cells. The GCT02 monoclonal IgG antibody was used to label three human glioma cell lines **(a)** U87, **(b)** U118 and **(c)** U251 to determine non‐specific binding by flow cytometry. The reagent successfully detected EGFRvIII on **(d)** U87‐EGFRvIII, **(e)** U118‐EGFRvIII and **(f)** U251‐EGFRvIII. Representative of at least three experiments. **(g)** Schematic representation of the second generation GCT02 and 2173 CAR constructs. Detection of the GCT02 (αMYC‐tag) and 2173 (αFLAG‐tag) CAR 6 day post‐transduction on the surface of **(h)** CD8^+^ **(i)** CD4^+^ primary human T cells by flow cytometry. A representative plot from one human donor is shown. Average transduction efficiencies of the GCT02 and 2173 CARs into human **(j)** CD8^+^ **(k)** CD4^+^ T cells as determined by MYC/FLAG tag labelling. Each symbol represents one individual transduction of one of four individual human donors. Shown is mean ± SD.

### The GCT02 CAR is specific for EGFRvIII and highly expressed in human T cells

EGFRvIII is a form of amplified EGFR, and it is well‐understood in the field that EGFR amplifications are poorly maintained on cells in culture.^22^ Given this poor maintenance of endogenous EGFRvIII expression, we previously described the generation of human glioma cell lines which were transduced with a lentiviral vector to express EGFRvIII stably, without requiring antibiotic selection.^18^ Having determined the GCT02 contact residues on EGFRvIII, we aquired a expanded library of human glioma cell models (Figure 2a–c) and created transduced EGFRvIII‐expressing variants (Figure 2d–f) which were labelled with the GCT02 IgG1 monoclonal reagent. Whilst a small shift in fluorescence was observed with parental cells labelled with GCT02, the cell lines transduced with EGFRvIII showed an approximate two‐log increase in fluorescence. This provided further confirmation of the GCT02 binder specificity.

A CAR was subsequently designed with the GCT02 binding domain. A benchmark for the function of this GCT02 CAR was generated using the EGFRvIII‐specific 2173 binding domain, designed by the University of Pennsylvania.^14^ To generate the human CAR constructs, the GCT02 or 2173 CAR, from the binding domain to CD3*ζ*, were cloned from the pMSCV‐CAR‐IRES‐mCherry vector^18^ into the pRRL‐SIN lentiviral vector; modified with an EF‐1*a* promoter (Figure 2g).

The CAR constructs were introduced via lentiviral transduction into primary human T cells. The CAR cell surface expression on CD8^+^ and CD4^+^ T cells was directly measured via flow cytometry by labelling with an anti‐MYC‐tag (GCT02) or anti‐FLAG‐tag (2173) antibody (Figure 2h and i, Supplementary figure 3). The pooled transduction efficiencies for each CAR were similar across multiple independent transductions of different PBMC donors (Figure 2j and k), although CD4^+^ T cells were generally transduced at a higher efficiency. Having confirmed CAR expression at the surface of the primary human T cells, the *in vitro* CAR function could be assessed.

### GCT02 human CAR T cells demonstrate selective and comparable cytotoxicity *in vitro*

**Figure 3:**
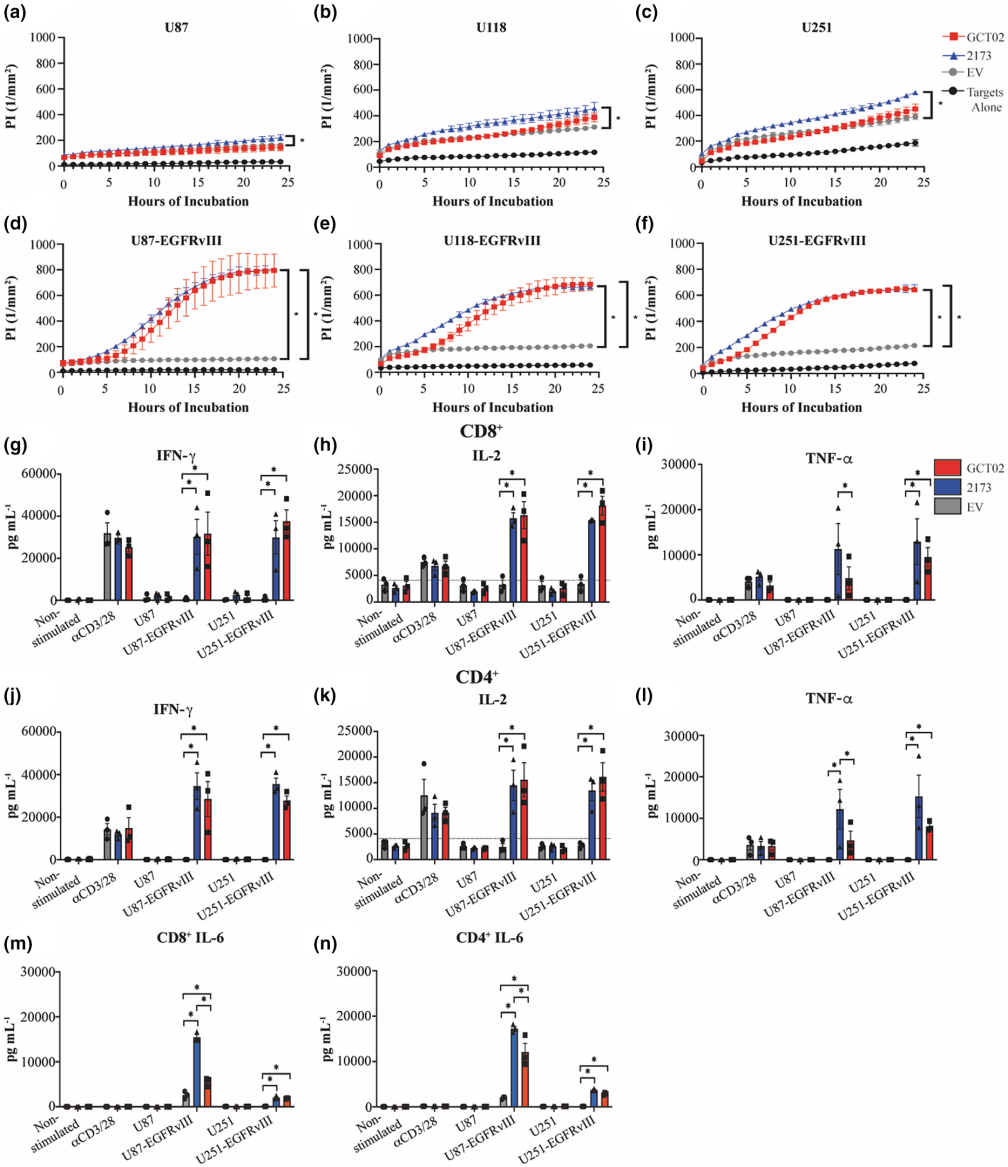
GCT02 CAR T cells demonstrate cytotoxic and cytokine‐secreting equivalence to the 2173 CAR. EGFRvIII‐specific CAR T cells were cocultured with three human glioblastoma parental cell lines **(a)** U87, **(b)** U118 and **(c)** U251 and the three EGFRvIII‐expressing variants **(d)** U87‐EGFRvIII, **(e)** U118‐EGFRvIII and **(f)** U251‐EGFRvIII. Cytotoxicity was measured kinetically using the IncuCyte platform over 24 h, and cell death indicated by uptake of propidium iodide. Shown is mean ± SD of triplicate measures, from one representative donor from three independent human donors. Statistical analysis by the unpaired *t*‐test, * *P‐*value < 0.05. CD8^+^ T cell secretion of **(g)** IFN‐γ, **(h)** IL‐2 and **(i)** TNF‐α. CD4^+^ T cell secretion of **(j)** IFN‐γ, **(k)** IL‐2 and **(l)** TNF‐α. **(m)** CD8^+^ and **(n)** CD4^+^ T cell secretion of IL‐6. All cytokine secretion was measured by cytokine bead array after an 18‐h coculture and T cell stimulation provided through αCD3/CD28 coated Dynabeads®. **(g–l)** The mean ± SEM from three independent human donors. **(m,n)** The mean ± SD from one human donor measured in triplicate. The dashed line in IL‐2 secretion indicates approximate background level of IL‐2 from cell culture medium. Statistical analysis by two‐way ANOVA, * *P‐*value < 0.05.

To investigate the cytotoxicity of the GCT02 CAR T cells, the IncuCyte microscopy‐based assay facilitated the kinetic analysis of tumor cell death. The uptake of the nuclear counterstain propidium iodide (PI) by glioma cells was considered a surrogate marker for cell death. The GCT02 and 2173 CAR T cells were cultured at a 1:1 effector‐to‐target ratio in the presence of either the parental or EGFRvIII‐expressing glioma cell line panel (Figure 3a–f) for 24 h and the number of PI‐positive cells was quantitated. Empty vector T cells (EV) were included as a negative control and used to determine the alloreactivity of the human T cells to the tumor cells. We did not observe any statistical difference in cytotoxicity between the GCT02 and 2173 CAR T cells, including at the other examined effector‐to‐target ratios (1:2, 2:1, Data not shown). GCT02 (and 2173) CAR T cell–mediated potent and equivalent EGFRvIII‐dependent cytotoxicity over the 24‐h coculture.

### GCT02 CAR T cells secrete pro‐inflammatory cytokines *in vitro*

The secreted cytokine profile may be an important predictor of potential inflammatory adverse effects. The GCT02 and 2173 CD8^+^ CAR and EV T cells from three independent PBMC donors were cocultured with positive control (TCR) ligand anti‐CD3/CD28 coated Dynabeads®, parental or EGFRvIII‐expressing U87 and U251 glioma cells for 18 h and the coculture supernatant probed for secretion of key effector cytokines IFN‐γ, IL‐2 and TNF‐α (Figure 3g–i).

All three T cell groups demonstrated the potential for cytokine secretion as determined by response to bead stimulation, and the cytokine levels induced by CAR stimulation were comparable between EGFRvIII‐expressing cell lines. GCT02 and 2173 CAR T cells secreted IFN‐γ (Figure 3g) and IL‐2 (Figure 3h) equivalently and specifically in response to EGFRvIII‐expressing tumor cell lines. However, the level of TNF‐α was more variable between PBMC donors (Figure 3i) and lower than the levels of IFN‐*γ* and IL‐2. The IFN‐γ cytokine secretion in response to CAR stimulation was like that induced by TCR stimulation; however, CAR‐mediated activation drove elevated IL‐2 levels compared with the TCR stimulation.

The secreted cytokine profile of the CD4^+^ CAR T cells (Figure 3j–l) indicated similar levels of IFN‐γ, and IL‐2 secretion by GCT02 and 2173 CAR T cells; however, there was reduced secretion of TNF‐α in response to the U87‐EGFRvIII cell line (Figure 3l) by GCT02 compared with 2173 CAR T cells.

Finally, we measured the secretion of the potent proinflammatory cytokine IL‐6 which has been implicated in cytokine release syndrome in haematological malignancies.^23^ We observed that IL‐6 was secreted specifically by both CD8^+^ and CD4^+^ CAR T cells in response to stimulation through the CAR by tumor cells expressing the EGFRvIII mutation (Figure 3m and n). There was statistically significantly lower IL‐6 secretion by GCT02 CAR T cells, compared with 2173 CAR T cells, by both CD4^+^ and CD8^+^ T cells against the U87‐EGFRvIII cell line.

### GCT02 CAR T cells engraft and accumulate at the tumor site to mediate regression of orthotopic models of glioblastoma

**Figure 4:**
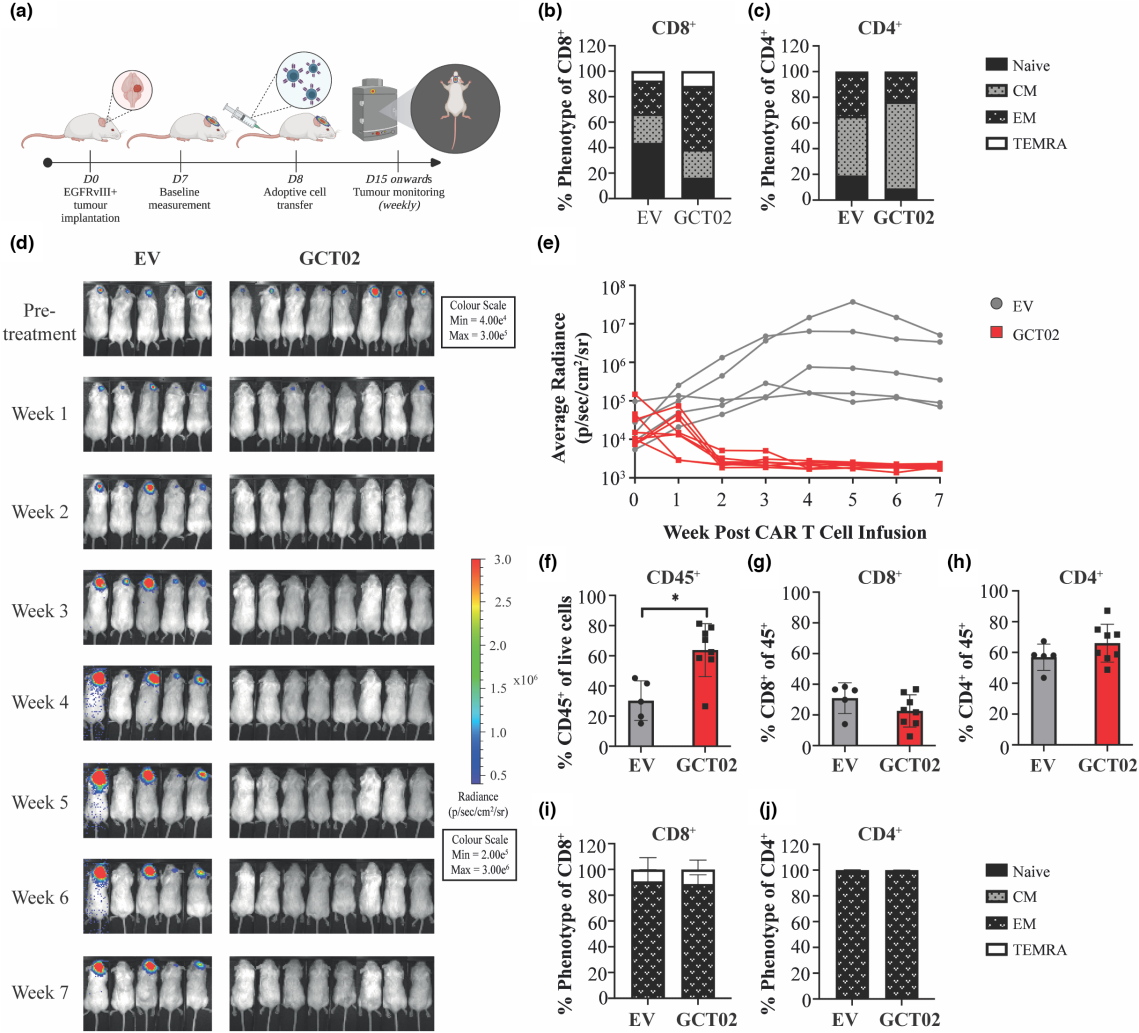
GCT02 CAR T cells mediate clearance and engraft in tumor‐bearing mice. **(a)** Schematic representation of *in vivo* intracranial experiments. EGFRvIII‐positive tumor cells were implanted into mice and after 1 week, tumor size was determined by bioluminescence imaging and mice were allocated to treatment groups. Mice received one infusion of CAR T cells via the tail vein and tumor size was monitored weekly. Pre‐infusion the **(b)** CD8^+^ and **(c)** CD4^+^ CAR T cells were phenotypically profiled by flow cytometry. **(d)** Bioluminescence images of mice bearing U251‐EGFRvIII tumors pre and up to 7‐week post‐CAR T cell treatment. The experiment was performed once with *N* = 5 mice in EV group, eight mice in GCT02 group. **(e)** Quantification of the tumors in mice shown in **d**. Each line represents a single mouse. At the experimental endpoint, the spleens of the mice were harvested and analysed by flow cytometry to determine the percentage of **(f)** CD45^+^ cells, **(g)** CD8^+^ and **(h)** CD4^+^ T cells. Shown is mean ± SD, each symbol is representative of one mouse. Statistical analysis by the unpaired *t*‐test, * *P‐*value < 0.05. Phenotypic analysis was performed on **(i)** CD8^+^ and **(j)** CD4^+^ T cells from the spleen 7‐week post‐infusion in the 5 EV‐treated mice and 8 GCT02‐treated mice. Shown is mean ± SD.

Having established the functional capabilities of our GCT02 CAR *in vitro*, it was important to demonstrate its ability to clear EGFRvIII‐expressing tumors *in vivo*. For this purpose, we utilised a previously described orthotopic xenograft model (Figure 4a), implanting U251‐EGFRvIII glioblastoma cells into the brains of NOD.Cg‐Prkdc^scid^IL2rg^tmWjl^/SzJ (NSG) mice. These cells express mCherry‐firefly luciferase enabling progressive monitoring of tumor size *in vivo* using bioluminescence imaging.

Pre‐infusion, the CD8^+^ (Figure 4b) and CD4^+^ (Figure 4c) CAR T cells were phenotyped by flow cytometry, evaluating the expression of markers CD45RO and CD197 (CCR7) (Supplementary figure 4). The CD8^+^ EV and GCT02 T cells demonstrated an expected predominantly effector memory phenotype, given the T cells were 16 days of post activation (Figure 4b). Smaller populations of the other T cell subsets were detected, although a larger population of naïve cells was evident in the EV group whilst a larger proportion of effector memory cells were detected in the GCT02 group. The CD4^+^ T cells demonstrated a skewing towards a central memory phenotype in both groups. Smaller populations of effector memory compared with the CD8^+^ T cells and an absence of a TEMRA population were observed (Figure 4c). The tumor‐bearing mice were treated with a single infusion of GCT02 or EV T cells at a 1:1 CD4^+^:CD8^+^ ratio, and tumor size was monitored weekly (Figure 4d). Two‐week post‐treatment, the tumors in GCT02‐treated mice were undetectable (Figure 4e). This apparent tumor clearance was maintained for 7 weeks, whilst tumors in EV‐treated mice grew. A splenic harvest indicated the transplanted CD45^+^ cells were detected in both groups, although a significantly higher percentage was detected in GCT02 than in EV‐treated mice (Figure 4f). Further analysis revealed engrafted CD8^+^ (Figure 4g) but predominantly CD4^+^ (Figure 4h) T cells.

Phenotypically, the engrafted T cells from both the GCT02 and the EV mice had differentiated to a near total effector memory state (Figure 4i and j) except for a small but relatively equivalent population of TEMRA cells in the CD8^+^ compartment.

**Figure 5:**
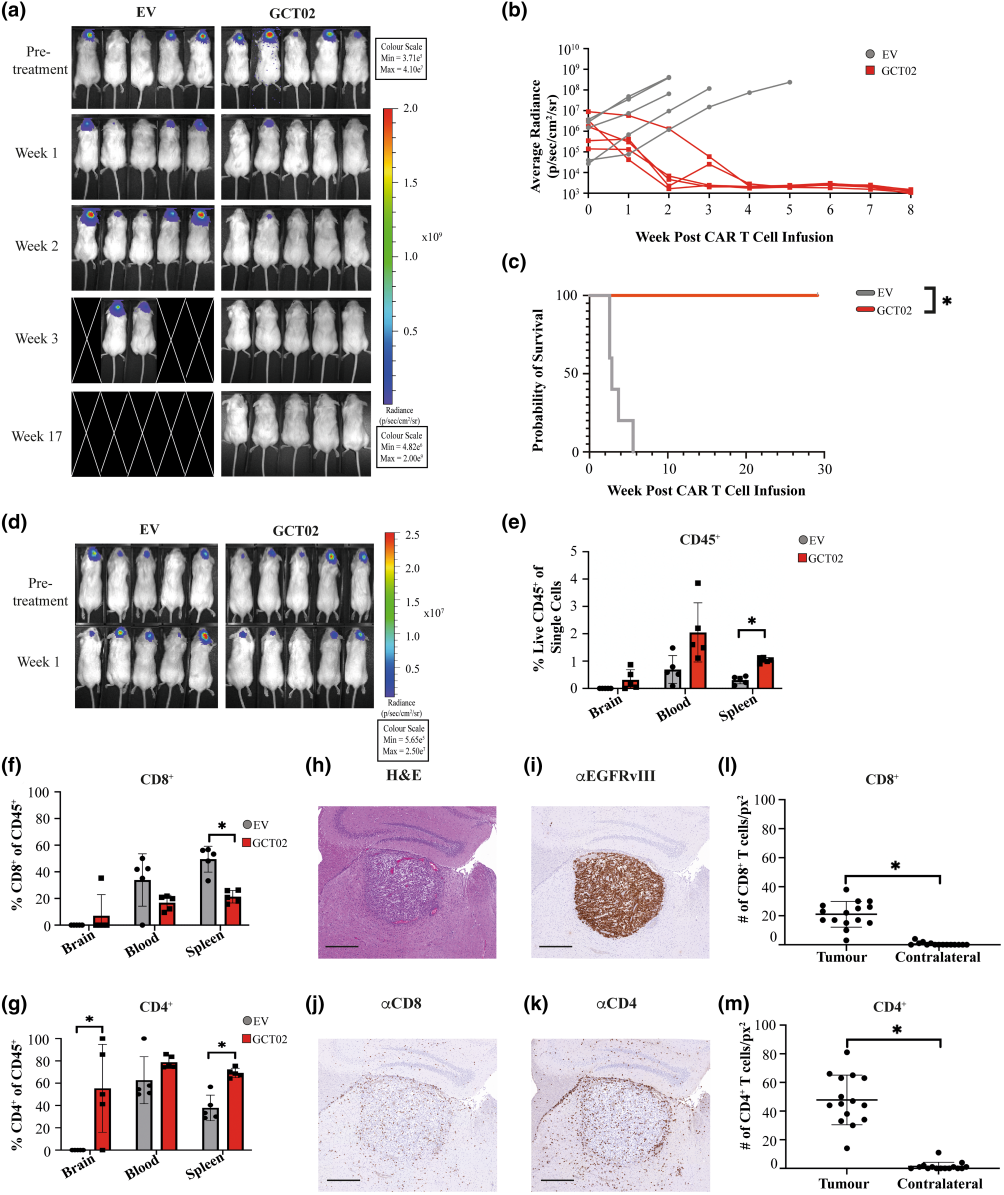
GCT02 CAR T cells infiltrate and clear U87‐EGFRvIII intracranial tumors. **(a)** Bioluminescence images of mice bearing U87‐EGFRvIII tumors pre and post CAR T cell treatment. This experiment was performed once, with the same human donor used in the experiment shown in Figure 4. *N* = 5 mice per group. **(b)** Quantification of the tumors in mice shown in **a**, where each line represents a single mouse. **(c)** Kaplan–Meier survival curve of the mice is shown in **a** and **b**. * *P*‐value < 0.05. **(d)** Bioluminescence images of mice bearing U87‐EGFRvIII tumors pre‐ and 1 week post‐CAR T cell treatment, using an independent human donor. This timed cull experiment was performed once with *N* = 5 mice per group. Flow cytometric analysis of brain, blood and spleen of T cell treated U87‐EGFRvIII treated mice identifying **(e)** CD45^+^ **(f)** CD8^+^ and **(g)** CD4^+^ human T cells. Each symbol represents a single mouse, shown as mean ± SD. Statistical analysis by the unpaired *t*‐test, * *P*‐value < 0.05. Brain sections from a cohort of U87‐EGFRvIII tumor bearing mice were stained 1 week post‐CAR T cell treatment with **(h)** Haematoxylin and Eosin, and antibodies specific for **(i)** EGFRvIII, **(j)** CD8 and **(k)** CD4. Images in **h–k** are also shown in Supplementary figure 7**a**. Scale bar is 500 μm and applies to all images. The infiltration of **(l)** CD8^+^ and **(m)** CD4^+^ human T cells was quantified in the tumor‐bearing and contralateral hemispheres. Shown is total mean ± SD, each symbol represents the numbers of T cell per square pixel, from five selected regions from three independent mice. Regions as demonstrated in Supplementary figure 8. Statistical analysis by the unpaired *t*‐test, *P‐value < 0.05.

Given the success of the GCT02 CAR T cells in this model, we sought validation in a second orthotopic glioblastoma model using the U87‐EGFRvIII cell line. The mice were implanted with tumors and treated with one T cell infusion (GCT02 CAR or EV) at 1:1 CD4^+^:CD8^+^. Tumor size was monitored weekly by bioluminescence imaging (Figure 5a).

Two‐weeks postinfusion, 80% of GCT02 CAR T cell–treated mice showed no detectable tumor, and by 4 weeks post‐treatment, all mice were cured (Figure 5b). All mice continued to survive to approximately 30 weeks post tumor cell implantation (Figure 5c) with no neurological or physical symptoms, at which the experiment was ended. Over the course of the experiment, an analysis of the peripheral blood samples from the GCT02‐treated mice showed that the infused CD8^+^ and CD4^+^ human T cells remained detectable over this time period (Supplementary figure 5). Next, we investigated CAR T cell infiltration into the U87‐EGFRvIII tumor 1‐week post‐treatment (Figure 5d). At this fixed time point, human CD45^+^ T cells could be identified in the blood, spleen and infiltrating the brain of CAR T cell–treated mice (Figure 5e). Of this population, whilst there were detectable CD8^+^ T cells in all three examined tissues (Figure 5f), a higher percentage of CD4^+^ T cells were found in mice treated with GCT02 CAR T cells compared with EV (Figure 5g), and statistically significantly increased in the brain and the spleen. Whether the improved CD4^+^:CD8^+^ CAR T cell ratio reflects increased brain penetrance or survival is unknown.

In a parallel cohort of GCT02‐treated mice, brain pathology defined tumor burden by histological analysis (Figure 5h). An EGFRvIII‐specific antibody (Supplementary figure 6) was used to examine the expression of the EGFRvIII protein 1 week post‐CAR T cell infusion. Within the tumors, EGFRvIII expression was highly maintained 1‐week post‐treatment (Figure 5i, Supplementary figure 7). Interestingly, CD8^+^ T cells were detectable using histology (Figure 5j, Supplementary Figure 7) but minimally detected by flow cytometry. A large accumulation of CD4^+^ T cells both within and surrounding the tumor site in GCT02‐treated mice (Figure 5k, Supplementary figure 7) was observed.

T cell infiltration was enumerated from five independent regions in the tumor‐bearing and contralateral hemispheres (Supplementary figure 8). Both CD8^+^ (Figure 5l) and CD4^+^ (Figure 5m) CAR T cells demonstrated increased infiltration into the tumor bearing compared with contralateral brain hemispheres, with greater infiltration of CD4^+^ T cells, supporting the flow cytometry results. GCT02 CAR T cells were functional *in vivo* and specifically trafficked to the tumor site. The final aspect to evaluate was the CAR T cell reactivity to healthy tissues as a more physiologic examination of the specificity of the GCT02 CAR.

### The GCT02 CAR demonstrates favorable target selectivity *in vitro*

**Figure 6:**
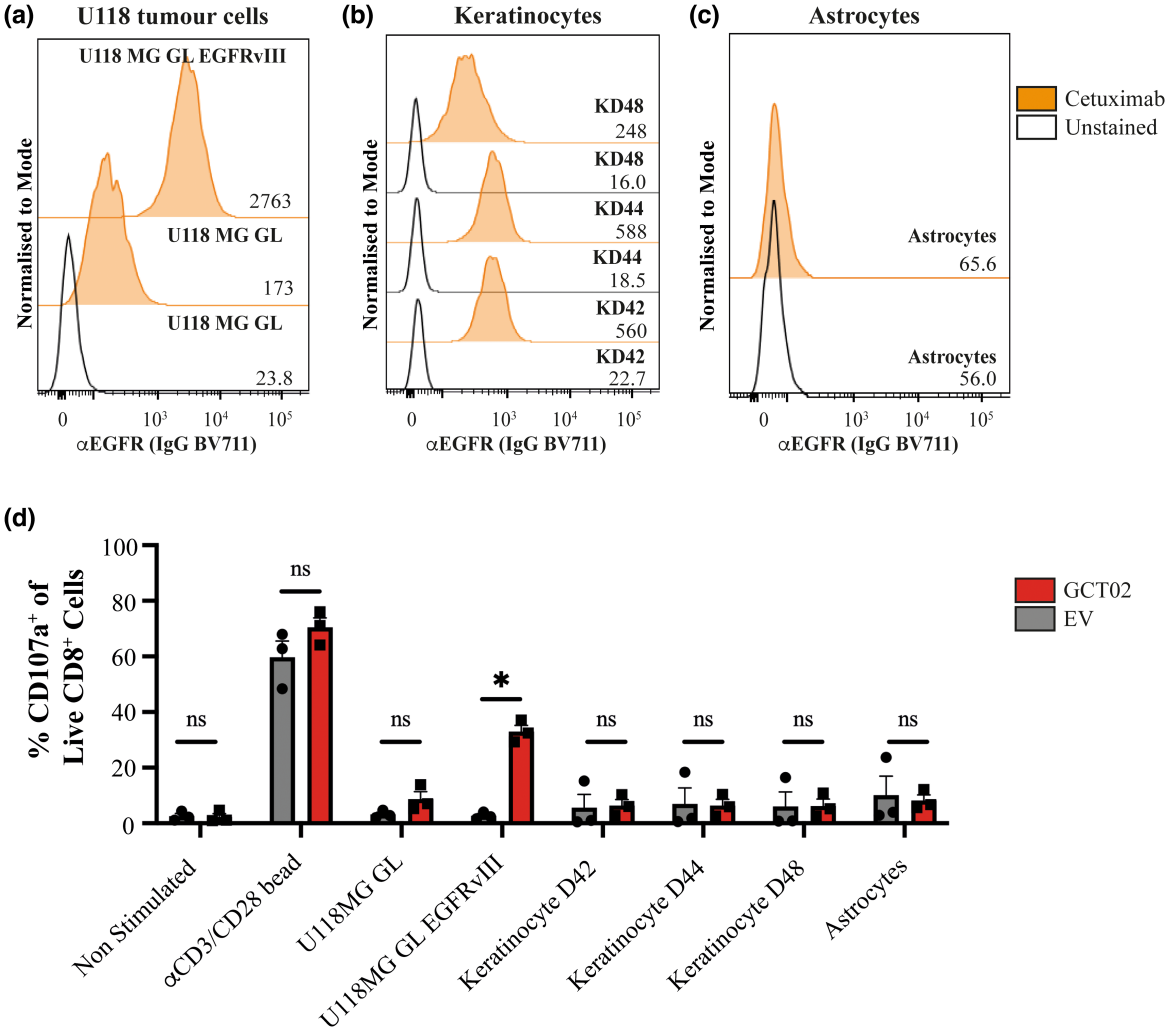
GCT02 CAR T cells are unreactive to primary EGFR‐expressing cells. The expression of EGFR was determined by flow cytometry via labelling with Cetuximab on **(a)** the human glioma cell line U118 and U118‐EGFRvIII **(b)** three donors of primary human keratinocytes and **(c)** primary human astrocytes. Values on the right of the plot denote the geometric mean fluorescence intensity. This experiment was performed once. **(d)** The degranulation of human T cells was measured by the exposure of CD107a after 4‐h coculture with different stimuli. Shown is mean ± SEM, each symbol represents the mean of the triplicate measures from one of three human donors. Statistical analysis by two‐way ANOVA, * *P*‐value < 0.05.

Target specificity is one of the greatest considerations for the development of precision‐based medicines; therefore, clinical translation of GCT02 CAR T cells required demonstration of a high target selectivity. We evaluated the selectivity of human GCT02 CAR T cells using a degranulation assay against human target cells with known EGFR/vIII status, determined from Cetuximab labelling (Figure 6a–c). The EGFRvIII mutation is caused by the truncation of the EGFR protein; therefore, binders to EGFRvIII may also bind EGFR. Our epitope mapping revealed GCT02 to bind to a shared region of EGFR and EGFRvIII, further emphasising the need to determine the propensity for EGFR‐related CAR T activation. Epidermal growth factor receptor is highly expressed in primary human keratinocytes (Human Protein Atlas, Figure 6b), and patients receiving EGFR‐targeted therapy have reported skin irritation.^24^ Keratinocytes were therefore selected as a critical cell type for screening. The primary human astrocytes were utilised as they would indicate GCT02 CAR T cell responsiveness to healthy brain cells.

To validate the EGFR status of the primary human cells, the keratinocytes and astrocytes were labelled with Cetuximab, and the expression of EGFR was determined by flow cytometry (Figure 6b and c, Supplementary figure 9). The keratinocytes highly expressed EGFR whilst the astrocytes did not (Figure 6b and c). Importantly, the U118 tumor target cells display robust levels of WT EGFR (Figure 6a), and coculture with the U118‐EGFRvIII cells resulted in a highly specific activation of the GCT02 CAR T cells (Figure 3e), therefore, this cell line made excellent positive and negative controlled targets for the specificity screen.

The functional consequences of a coculture of the GCT02 CAR T cells with healthy human keratinocyte and astrocyte cells was determined by T cell degranulation, indicated by CD107a exposure on the T cells as measured by flow cytometry (Supplementary figure 10). CAR T cells generated from three independent PBMC donors were cocultured in triplicate with either *α*CD3/CD28 Dynabeads®, the U118 cell line panel or primary cells for 4 h in the presence of the anti‐CD107a antibody, a surrogate for T cell degranulation and inferred cytotoxicity. Despite the very high effector‐to‐target ratio of 1:1 (relative to physiological levels), the GCT02 CAR T cells did not expose CD107a to a greater extent than the EV T cells in any condition other than with the positive control U118‐EGFRvIII cells (Figure 6d). There was no response to healthy brain astrocytes. Considering these data, the GCT02 CAR can be described as possessing a high selectivity to the EGFRvIII mutation over the endogenous EGFR protein which is likely to be clinically favorable.

## Discussion

Glioblastoma is the most aggressive form of adult brain tumor. Today, diagnosed patients are given the same 5‐year overall survival rate as three decades ago, a dismal 5%. The current treatment protocols are ineffective at achieving complete, long‐term regression, given the diffuse and resistant nature of the tumor. Glioblastoma relapse is almost inevitable, and survival is poor.^25^ The tumor subclones responsible for relapse are hypothesised to genetically diverge early on in tumor development and reside in nonresected brain regions,^26^ strongly emphasising the critical need for effective frontline therapies.

CAR T cells have been highly effective in treating liquid malignancies, primarily through targeting the pan B‐cell marker CD19.^27^, ^28^, ^29^ Target antigen selection in many solid tumors is challenging,^30^ because of the limited number of tumor‐specific markers identified. Additionally, there is an increased risk of targeting proteins in the brain with a shared expression on healthy and malignant cells. Whilst not necessarily considered a prognostic marker,^31^ the EGFRvIII mutation reportedly confers radio‐^32^ and chemotherapy^33^ resistance. Critically, EGFRvIII expression is restricted to glioblastoma cells, presenting a tumor‐specific target for CAR T cell therapy for 25–30% of glioblastoma patients. Whilst EGFRvIII CAR T cell monotherapy has failed partly because of antigen escape,^16^ there is utility in identifying novel binders to utilise in logic‐gated approaches, confining therapeutic approaches precisely at the tumor site.

To date, the EGFRvIII‐binders in CAR T cell products have been generated by the rederivation of pre‐existing monoclonal antibodies. We recently published a *de novo* generated binder, with an approximate 300‐fold higher affinity than the reported affinity of the 2173 binder. Whilst the CAR T cell field is looking to detune affinity to improve antigen discrimination,^34^ we demonstrated that higher affinity constructs do not impede effective CAR function. Both the murine and human GCT02 CAR T cells secrete lower quantities of pro‐inflammatory cytokines whilst maintaining the capacity to mediate robust cytotoxic and *in vivo* antitumor responses.^18^

The DMS study determined whilst GCT02 binds a shared EGFR/vIII region (Figure 1), our functional studies demonstrated EGFRvIII specificity. We speculate the specificity is driven not through a unique EGFRvIII epitope, but steric mechanisms because of differences in orientation, conformation or differential glycosylation of EGFR and EGFRvIII at the cell membrane.

The cytokine bead array assays enabled the comparison of selected secreted cytokines between CAR constructs. Despite the input cell number being normalised for CAR expression, and therefore higher total cell numbers with the capacity to respond to the T cell stimulation, two of three investigated effector cytokines were secreted at a lower level in response to TCR stimulation than CAR stimulation. This suggests the GCT02 CAR is potent, demonstrating high production of pro‐inflammatory cytokines such as IFN‐γ and IL‐2, critical to the development of cytotoxic T cells.^35^ Importantly, however, the GCT02 CAR T cell secretion of IL‐6 against the U87‐EGFRvIII cell line was significantly reduced compared with the 2173 CAR T cell benchmark, a potential indicator of a lower inflammatory profile.

The GCT02 CAR T cells demonstrated curative *in vivo* efficacy in two orthotopic murine models. A single infusion was sufficient to induce regression of existing intracranial tumors, and 7 week post‐treatment engrafted GCT02 CAR T cells could be detected in the spleens of treated mice. GCT02 CAR treatment was shown to protect the mice from the tumor for as long as 30‐weeks post‐therapy. The differentiation of infused cells from both treatment groups to a mostly effector‐like state suggests this is unlikely because of exposure to antigen or function, but rather a consequence of T cell age and *in vivo* infusion.

The immunotherapy field is certainly turning its attention to dissecting the potentially variable roles of CD4^+^ and CD8^+^ CAR T cells in the antitumor response. Preclinically, a study by Wang *et al*.^36^ reported CD4^+^ CAR T cells showed greater persistence and potency than CD8^+^ CAR T cells. Additionally, other groups are investigating how the CAR T manufacturing process may influence and tailor different T cell subsets and their subsequent function. Subset importance has been recognised clinically, with a defining feature of the FDA‐approved product Lisocabtagene Maraleucel (Breyanzi), the defined ratios of CD4^+^:CD8^+^ CAR T cells.^37^

Here, despite injecting a 1:1 ratio of CD4^+^ to CD8^+^ CAR T cells, a higher CD4^+^:CD8^+^ T cell ratio was detected intratumorally 1 week after treatment (Figure 5l and m). Whether the discordant CD4^+^:CD8^+^ T cell ratio is because of enhanced CD4^+^ CAR T cell proliferation, or poor CD8^+^ CAR T cell survival, remains to be elucidated. Future studies in CAR T cell therapy for brain malignancies may benefit from elucidating the differential proliferative potential and persistence of CD4^+^ and CD8^+^ CAR T cell subsets.

The specificity of any novel CAR T cell product is of utmost importance for clinical translation. As the DMS indicated binding to a shared EGFR/vIII region, it was critical to evaluate the binder for the potential for cross‐reactivity to EGFR. EGFR‐related toxicity has been reported using *in vitro* and *in vivo* methods.^14^ Facilitating clinical translation, our *in vitro* screen showed little reactivity of the GCT02 CAR to three independent keratinocyte donors and primary astrocytes, even at the much higher effector‐to‐target ratio of 1:1 than what would be encountered physiologically. The selectivity of the GCT02 CAR T cells for EGFRvIII stimulation was clear and suggests a favorable selectivity profile for translation.

There can be no doubt that glioblastoma poses considerable therapeutic challenges. The brain is delicate, consisting of regions critical to human function and survival. All efforts should be made to translate safe and highly effective therapies. These data provide evidence of a highly functional and specific EGFRvIII CAR construct which may be beneficial for patients with glioblastoma. Future studies translating GCT02 CAR T cells in combination with logic‐gated engineered CAR T cells or with other therapeutics will be the focus of future translation.

The novel GCT02 CAR demonstrates EGFRvIII‐specific potent cytotoxicity and cytokine capacity. Moreover, the GCT02 CAR T cells were functional in two *in vivo* models, infiltrating the tumor site to mediate regression. We report no significant cross‐reactivity of GCT02 to EGFR protein, demonstrating a highly specific new binder for glioblastoma.

This study provides the preclinical data package serving as the launchpad for a clinical trial to test GCT02 CAR T cell safety and efficacy in humans.

## Methods

### Cell lines and cell culture

Human glioblastoma cell lines: U87 (Luwor Laboratory, Royal Melbourne Hospital, Melbourne, Australia), U118 (American Type Culture Collection, ATCC) were transduced to express truncated nonsignalling EGFRvIII as well as GFP Firefly luciferase (pFUGW‐Luc‐T2A‐GFP) as previously described.^18^ The U251‐mCherry‐Luciferase cell lines were a kind gift from the Strasser Laboratory (Walter and Eliza Hall Institute, Melbourne, Australia). Cells were maintained RPMI (Walter and Eliza Hall Institute, or Gibco, Life Technologies, Waltham, USA) supplemented with 10% Heat‐Inactivated Foetal Calf Serum (FCS) (Sigma Aldrich, St Louis, USA), 100 U mL^−1^ Penicillin & 100 mg mL^−1^ streptomycin (Pen/Strep). BW5147 (Call Laboratory, Walter and Eliza Hall Institute, Australia) and primary T cells (Australian Red Cross Agreement Number 21‐07VIC‐12) were maintained in RPMI media (Gibco or Walter and Eliza Hall Institute, Australia) supplemented with 10% FCS (Sigma Aldrich), 2 mm Glutamax, Pen/Strep, 0.1 mm MEM nonessential amino acids, 10 mm HEPES buffer solution, 1 mm sodium pyruvate, 5 0 mm 2‐mercaptoethanol. Primary T cells were cultured with 50 IU mL^−1^ rhIL‐2 (Peprotech, Cranbury, USA). PBMC were isolated by density centrifugation before T cells were isolated with EasySep kits following the manufacturer's instructions (StemCell Technologies, Vancouver, Canada). All cell lines were maintained at 37°C, 5% CO_2_.

### Primary keratinocytes and astrocytes

Primary human keratinocytes (three donors, PrimaCyt, Schwerin, Germany) and astrocytes (one donor, growth media, Neuromics, Edina, USA) were thawed immediately before use. Keratinocyte media recipe kindly provided by Dr Vaughan Feisst (University of Auckland, New Zealand).

### Genetic constructs

Chimeric antigen receptor vectors were generated using Gibson Assembly cloning from the previously described GCT02 and 2173 vectors^18^ into a modified lentiviral transfer vector pRRL‐SIN‐WPRE‐GFP (Addgene #12252), replacing WPRE with EF‐1α minimal promoter and GFP with CAR. The EGFRvIII nonsignalling construct was previously described.^18^

### Flow cytometry

Antigen expression was detected using 5 μg/mL of GCT02 IgG (ATUM, Newark, USA) or 5 μg mL^−1^ of Cetuximab (Erbitux, Merck Serono, Darmstadt, Germany) and mouse anti‐human IgG‐BV711 (Becton Dickenson Biosciences, New Jersey, USA) secondary. To determine CAR cell surface expression, *α*MYCtag or *α*FLAG‐tag, *α*CD3, *α*CD4, *α*CD8 antibodies were used (refer to Supplementary table 1). For phenotyping, T cells were labelled with Fixable Yellow (Invitrogen, Waltham, USA), *α*CD45, *α*CD4, *α*CD8, *α*CD45RO and *α*CD197 antibodies (Supplementary table 1). CAR T cell degranulation was measured by coculture of three independent PBMC donors at a 1:1 ratio with *α*CD3/CD28 Dynabeads® (Life Technologies, Carlsbad, USA), U118/EGFRvIII, primary keratinocytes or astrocytes and the *α*CD107a‐PE antibody (Becton Dickenson Pharmingen) at 37°C, 5% CO_2_ for 4 h. Cells were labelled with a *α*CD8 antibody (Supplementary table 1), before washing and adding DAPI before flow cytometry analysis, using a Becton Dickenson FortessaX20 flow cytometer and FlowJo software (Becton Dickenson, version 10.8).

### T cell transduction

HEK293T cells were transfected with lentivirus packaging and transfer vectors using Fugene (Promega, Madison, USA) reagent following the manufacturer's protocols (Promega, Madison, USA). Purified T cells were stimulated with αCD3/CD28 Dynabeads® (Life Technologies) for 48 h before removal. Lentiviral spinoculation was performed 48‐ and 72‐h postactivation using retronectin‐coated plates (Takara Bio, Kusatsu, Japan).

### Deep mutational scanning

A library of 3000 DNA variants of truncated EGFRvIII (residues 1–380) was constructed targeting residues 1–43 and solvent‐exposed residues 44–348 of EGFRvIII. The library was synthesised by Twist Biosciences (South San Francisco, USA) and installed in the MMLV retroviral Gateway vector pMX‐GW‐PGK‐PuroR‐GFP^38^, ^39^ (kind gift from Andrew Brooks, University of Queensland, Australia). Each variant was tagged with approximately 10 barcodes. Long‐read sequencing on a SMRTcell 8 m chip using a PacBio Sequel II was used to match each variant with its respective barcode (AGRF‐UQ). Three replicate libraries of virus containing pMX‐GW‐Hygro were produced to minimise barcode switching among variants in retroviral virions.^40^ Each library of variants was screened in BW5147 cells with puromycin selection and FACS (Becton Dickenson FACS ARIA III). Nonsorted cells from each BW5147 library were snap‐frozen to determine library diversity. Sorted populations were processed for mRNA using the Qiagen RNeasy kit (Qiagen, Hilden, Germany, Catalogue 74106) following the manufacturer's instructions. cDNA was amplified and indexed with three separate index primer pairs to allow demultiplexing after sequencing. Paired‐end sequencing of barcodes was performed on an Illumina NextSeq 1000/2000 cartridge with P1 reagents. Cutadapt v3.7 was used to demultiplex samples, and Read one and Read two were merged with USEARCH v9.2.64_i86linux32^41^ and further trimmed to the variant barcode with Cutadapt v3.7. Enrich2^20^ was used to calculate the change in variant frequencies among samples and identify predicted antibody epitopes. These were converted to pseudo‐B‐factors to colour the surface of an AlphaFold model of EGFRvIII.

### Cytotoxicity assay

T cell cytotoxicity was measured using the IncuCyte platform (Sartorius, Goettingen, Germany, Models S3, SX5), using propidium iodide (PI, 50 μm) to indicate cell death. Target cells were seeded the day before and CAR T cells added at 1effector:1target ratio normalised for CAR expression as determined by flow cytometry. Saponin (Sigma Aldrich) provided maximum PI reading. Analysis was performed using the IncuCyte software (Sartorius, version 2021A).

### Cytometric bead array

Chimeric antigen receptor T cells were assayed for cytokine secretion by culturing in media alone, with αCD3/CD28 Dynabeads® (Life Technologies) or 1:1 with antigen‐expressing cells, normalising for CAR expression. Cytokines IFN‐γ, TNFα, IL‐2 and IL‐6 were measured in the supernatant using Cytokine Bead Array (CBA) Flex sets (Becton Dickinson).

### Mice

Six‐ to eight‐week‐old NOD.Cg‐Prkdc^scid^IL2rg^tmWjl^/SzJ (NSG) (Charles River Gut Flora) female mice were bred under specific pathogen‐free conditions at WEHI Kew facility and maintained in the animal facility at WEHI (Parkville, Victoria, Australia) under WEHI AEC approval 2019.020.

### Xenograft models

Glioblastoma cells (5 × 10^4^) were intracranially implanted into mice as previously described.^18^ Following surgery, the mice were weighed and checked daily for 7‐day postsurgery. The mice were subsequently visually checked three times weekly and weighed once weekly until the conclusion of the experiment. Treatment groups were assigned based on tumor measurements biased against the experimental group, with 1 × 10^7^ CAR T cells (1CD4^+^:1CD8^+^) injected intravenously via the tail vein. For each *in vivo* experiment shown in Figure 5, CAR T cells were generated from a separate human donor.

Ethical endpoints for *in vivo* experiments were defined by approved ethical guidelines (WEHI AEC #2019.020). Mice were required to be euthanised if any of the following occurred; mice lost more than 20% of their presurgery weight, if mice exhibited either prolonged or severe neurological or physical dysfunction, such as circling or hunched body positions or if the surgical site became exposed.

### Bioluminescence imaging

Intracranial tumors were imaged using 3 mg VivoGlo Luciferin (Promega) IP injected into mice and IVIS Lumina III Series Hardware (Perkin‐Elmer, Waltham, USA). Image analysis was performed using Living Image Software (Perkin‐Elmer, version 4.7.2), and average radiance values (Radiance/cm/s/sr) were plotted in Graphpad Prism (Dotmatics, Boston, USA).

### Histology tissue preparation

Brains of mice bearing intracranial tumors were fixed for 48 h in 10% neutral buffered formalin. Tissue sections were paraffin‐embedded and sectioned. Sections were stained with Haematoxylin and Eosin (H&E), *a*CD4, *a*CD8 or anti‐EGFRvIII (LMH‐151)^42^ antibodies (Supplementary table 2). Anti‐rat and anti‐rabbit IgG conjugated to horseradish peroxidase were used for secondary binding (Supplementary table 2), and signal was developed with diaminobenzidine followed by counterstaining with haematoxylin. Slides were scanned using a PANORAMIC scan II (3DHISTECH, Budapest, Hungary).

### Histological sample quantification

T cell quantification was performed by randomly selecting five 1500‐pixel regions in tumor‐bearing and contralateral hemispheres (three mice). Using the QuPath StarDist function, CD4^+^ and CD8^+^ T cells were enumerated per region per hemisphere and count normalised to per million pixels. Results were filtered by eliminating objects < 611 px^2^ in size and objects where the DAB mean was < 0.118. Total T cells were determined from the addition of CD4 and CD8 infiltrations per hemisphere.

### Study design and statistical analysis

Experiments were performed multiple times in biological replicates as per figure captions. Data are presented as Mean ± SD, or as otherwise stated. Statistical analysis was performed using GraphPad Prism (version 8.4.3), and significance was determined from analysis as indicated in figure captions. Asterisks refer to *P* < 0.05.

## AUTHOR CONTRIBUTIONS

**Rebecca Abbott:** Formal Analysis; Investigation; Methodology; Resources; Validation; Vizualization; Writing‐original draft; Writing‐review and editing. **Melinda Iliopoulos, Katherine A Watson, Valeria Arcucci, Margareta Go, Hannah E Hughes‐Parry, Pete Smith, Melissa Call:** Resources; Methodology; Formal Analysis; Investigation; Resources; Validation; Vizualization; Writing‐review and editing. **Ryan S Cross:** Conceptualization; Data Curation; Resources; Methodology; Project Administration; Formal Analysis; Investigation; Resources; Supervision; Validation; Vizualization; Writing‐original draft; Writing‐review and editing. **Misty R Jenkins:** Conceptualization; Data Curation; Resources; Methodology; Project Administration; Formal Analysis; Funding Acquisition; Investigation; Resources; Supervision; Validation; Vizualization; Writing‐original draft; Writing‐review and editing.

## Conflict of interest

RCA, RSC and MRJ are listed as inventors on a patent filed with the GCT02 CAR presented here.

## Ethics approval

Animal experiments were conducted with the ethical approval of the WEHI animal ethics committee (2019.020). Human PBMCs were obtained from the Australia Red Cross (Agreement Number 21‐07VIC‐12).

## Supporting information


Supporting information

## Data Availability

The data that support the findings of this study are available from the corresponding author upon reasonable request.

## References

[1] Miller KD , Ostrom QT , Kruchko C *et al*. Brain and other central nervous system tumor statistics, 2021. CA Cancer J Clin 2021; 71: 381–406. 34427324 10.3322/caac.21693

[2] Brain and Other Central Nervous System Cancers. Canberra: Australian Institute of Health and Welfare; 2017:80.

[3] Stupp R , Mason WP , van den Bent MJ *et al*. Radiotherapy plus concomitant and adjuvant temozolomide for glioblastoma. N Engl J Med 2005; 352: 987–996. 15758009 10.1056/NEJMoa043330

[4] Daneman R , Prat A . The blood‐brain barrier. Cold Spring Harb Perspect Biol 2015; 7: a020412. 25561720 10.1101/cshperspect.a020412 PMC4292164

[5] van Tellingen O , Yetkin‐Arik B , de Gooijer MC , Wesseling P , Wurdinger T , de Vries HE . Overcoming the blood–brain tumor barrier for effective glioblastoma treatment. Drug Resist Updat 2015; 19: 1–12. 25791797 10.1016/j.drup.2015.02.002

[6] Sarkaria JN , Hu LS , Parney IF *et al*. Is the blood–brain barrier really disrupted in all glioblastomas? A critical assessment of existing clinical data. J Neurooncol 2018; 20: 184–191. 10.1093/neuonc/nox175 PMC5777482 29016900

[7] Bockmayr M , Klauschen F , Maire CL *et al*. Immunological profiling of mutational and transcriptional subgroups in pediatric and adult high‐grade gliomas. Cancer Immunol Res 2019; 7: 1401–1411. 31266783 10.1158/2326-6066.CIR-18-0939

[8] Kmiecik J , Poli A , Brons NHC *et al*. Elevated CD3^+^ and CD8^+^ tumor‐infiltrating immune cells correlate with prolonged survival in glioblastoma patients despite integrated immunosuppressive mechanisms in the tumor microenvironment and at the systemic level. J Neuroimmunol 2013; 264: 71–83. 24045166 10.1016/j.jneuroim.2013.08.013

[9] Ahmed N , Brawley V , Hegde M *et al*. HER2‐specific chimeric antigen receptor–modified virus‐specific T cells for progressive glioblastoma: A phase 1 dose‐escalation trial. JAMA Oncol 2017; 3: 1094–1101. 28426845 10.1001/jamaoncol.2017.0184 PMC5747970

[10] Brown CE , Badie B , Barish ME *et al*. Bioactivity and safety of IL13Rα2‐redirected chimeric antigen receptor CD8^+^ T cells in patients with recurrent glioblastoma. Clin Cancer Res 2015; 21: 4062–4072. 26059190 10.1158/1078-0432.CCR-15-0428 PMC4632968

[11] Yi Z , Prinzing BL , Cao F , Gottschalk S , Krenciute G . Optimizing EphA2‐CAR T cells for the adoptive immunotherapy of glioma. Mol Ther Methods Clin Dev 2018; 9: 70–80. 29552579 10.1016/j.omtm.2018.01.009 PMC5852415

[12] Moscatello DK , Holgado‐Madruga M , Godwin AK *et al*. Frequent expression of a mutant epidermal growth factor receptor in multiple human tumors. Cancer Res 1995; 55: 5536–5539. 7585629

[13] Faulkner C , Palmer A , Williams H *et al*. EGFR and EGFRvIII analysis in glioblastoma as therapeutic biomarkers. Br J Neurosurg 2015; 29: 23–29. 25141189 10.3109/02688697.2014.950631

[14] Johnson LA , Scholler J , Ohkuri T *et al*. Rational development and characterization of humanized anti‐EGFR variant III chimeric antigen receptor T cells for glioblastoma. Sci Transl Med 2015; 7: 275ra222. 10.1126/scitranslmed.aaa4963 PMC4467166 25696001

[15] Morgan RA , Johnson LA , Davis JL *et al*. Recognition of glioma stem cells by genetically modified T cells targeting EGFRvIII and development of adoptive cell therapy for glioma. Hum Gene Ther 2012; 23: 1043–1053. 22780919 10.1089/hum.2012.041 PMC3472555

[16] O'Rourke DM , Nasrallah MP , Desai A *et al*. A single dose of peripherally infused EGFRvIII‐directed CAR T cells mediates antigen loss and induces adaptive resistance in patients with recurrent glioblastoma. Sci Transl Med 2017; 9: eaaa0984. 28724573 10.1126/scitranslmed.aaa0984 PMC5762203

[17] Goff SL , Morgan RA , Yang JC *et al*. Pilot trial of adoptive transfer of chimeric antigen receptor‐transduced T cells targeting EGFRvIII in patients with glioblastoma. J Immunother 2019; 42: 126–135. 30882547 10.1097/CJI.0000000000000260 PMC6691897

[18] Abbott RC , Verdon DJ , Gracey FM *et al*. Novel high‐affinity EGFRvIII‐specific chimeric antigen receptor T cells effectively eliminate human glioblastoma. Clin Transl Immunol 2021; 10: e1283. 10.1002/cti2.1283 PMC8106904 33976881

[19] Li S , Schmitz KR , Jeffrey PD , Wiltzius JJW , Kussie P , Ferguson KM . Structural basis for inhibition of the epidermal growth factor receptor by cetuximab. Cancer Cell 2005; 7: 301–311. 15837620 10.1016/j.ccr.2005.03.003

[20] Rubin AF , Gelman H , Lucas N *et al*. A statistical framework for analyzing deep mutational scanning data. Genome Biol 2017; 18: 150. 28784151 10.1186/s13059-017-1272-5 PMC5547491

[21] Jumper J , Evans R , Pritzel A *et al*. Highly accurate protein structure prediction with AlphaFold. Nature 2021; 596: 583–589. 34265844 10.1038/s41586-021-03819-2 PMC8371605

[22] Bigner SH , Humphrey PA , Wong AJ *et al*. Characterization of the epidermal growth factor receptor in human glioma cell lines and xenografts. Cancer Res 1990; 50: 8017–8022. 2253244

[23] Maude SL , Barrett D , Teachey DT , Grupp SA . Managing cytokine release syndrome associated with novel T cell‐engaging therapies. Cancer J 2014; 20: 119–122. 24667956 10.1097/PPO.0000000000000035 PMC4119809

[24] Galizia G , Lieto E , De Vita F *et al*. Cetuximab, a chimeric human mouse anti‐epidermal growth factor receptor monoclonal antibody, in the treatment of human colorectal cancer. Oncogene 2007; 26: 3654–3660. 17530019 10.1038/sj.onc.1210381

[25] Stupp R , Hegi ME , Mason WP *et al*. Effects of radiotherapy with concomitant and adjuvant temozolomide versus radiotherapy alone on survival in glioblastoma in a randomised phase III study: 5‐year analysis of the EORTC‐NCIC trial. Lancet Oncol 2009; 10: 459–466. 19269895 10.1016/S1470-2045(09)70025-7

[26] Spiteri I , Caravagna G , Cresswell GD *et al*. Evolutionary dynamics of residual disease in human glioblastoma. Ann Oncol 2019; 30: 456–463. 30452544 10.1093/annonc/mdy506 PMC6442656

[27] Maude SL , Frey N , Shaw PA *et al*. Chimeric antigen receptor T cells for sustained remissions in leukemia. N Engl J Med 2014; 371: 1507–1517. 25317870 10.1056/NEJMoa1407222 PMC4267531

[28] Maude SL , Laetsch TW , Buechner J *et al*. Tisagenlecleucel in children and young adults with B‐cell lymphoblastic leukemia. N Engl J Med 2018; 378: 439–448. 29385370 10.1056/NEJMoa1709866 PMC5996391

[29] Locke FL , Ghobadi A , Jacobson CA *et al*. Long‐term safety and activity of axicabtagene ciloleucel in refractory large B‐cell lymphoma (ZUMA‐1): a single‐arm, multicentre, phase 1–2 trial. Lancet Oncol 2019; 20: 31–42. 30518502 10.1016/S1470-2045(18)30864-7 PMC6733402

[30] Abbott RC , Cross RS , Jenkins MR . Finding the keys to the CAR: identifying novel target antigens for T cell redirection immunotherapies. Int J Mol Sci 2020; 21: 515. 31947597 10.3390/ijms21020515 PMC7014258

[31] Thuy MN , Kam JK , Lee GC *et al*. A novel literature‐based approach to identify genetic and molecular predictors of survival in glioblastoma multiforme: analysis of 14,678 patients using systematic review and meta‐analytical tools. J Clin Neurosci 2015; 22: 785–799. 25698544 10.1016/j.jocn.2014.10.029

[32] Mukherjee B , McEllin B , Camacho CV *et al*. EGFRvIII and DNA double‐Strand break repair: a molecular mechanism for radioresistance in glioblastoma. Cancer Res 2009; 69: 4252–4259. 19435898 10.1158/0008-5472.CAN-08-4853 PMC2694953

[33] Nagane M , Levitzki A , Gazit A , Cavenee WK , Huang HJS . Drug resistance of human glioblastoma cells conferred by a tumor‐specific mutant epidermal growth factor receptor through modulation of Bcl‐XL and caspase‐3‐like proteases. Proc Natl Acad Sci USA 1998; 95: 5724. 9576951 10.1073/pnas.95.10.5724 PMC20446

[34] Liu X , Jiang S , Fang C *et al*. Affinity‐tuned ErbB2 or EGFR chimeric antigen receptor T cells exhibit an increased therapeutic index against tumors in mice. Cancer Res 2015; 75: 3596–3607. 26330166 10.1158/0008-5472.CAN-15-0159 PMC4560113

[35] Maraskovsky E , Chen WF , Shortman K . IL‐2 and IFN‐gamma are two necessary lymphokines in the development of cytolytic T cells. J Immunol 1989; 143: 1210–1214. 2501391

[36] Wang D , Aguilar B , Starr R *et al*. Glioblastoma‐targeted CD4^+^ CAR T cells mediate superior antitumor activity. JCI Insight 2018; 3: e99048. 29769444 10.1172/jci.insight.99048 PMC6012522

[37] Turtle CJ , Hanafi L‐A , Berger C *et al*. Immunotherapy of non‐Hodgkin's lymphoma with a defined ratio of CD8^+^ and CD4^+^ CD19‐specific chimeric antigen receptor‐modified T cells. Sci Transl Med 2016; 8: 355ra116‐355ra116. 10.1126/scitranslmed.aaf8621 PMC5045301 27605551

[38] Bridgford JL , Lee SM , Lee CMM *et al*. Novel drivers and modifiers of MPL‐dependent oncogenic transformation identified by deep mutational scanning. Blood 2020; 135: 287–292. 31697803 10.1182/blood.2019002561

[39] Chhabra Y , Wong HY , Nikolajsen LF *et al*. A growth hormone receptor SNP promotes lung cancer by impairment of SOCS2‐mediated degradation. Oncogene 2018; 37: 489–501. 28967904 10.1038/onc.2017.352 PMC5799715

[40] Feldman D , Singh A , Garrity AJ , Blainey PC . Lentiviral co‐packaging mitigates the effects of intermolecular recombination and multiple integrations in pooled genetic screens. bioRxiv 2018 262121. 10.1101/262121

[41] Edgar RC . Search and clustering orders of magnitude faster than BLAST. Bioinformatics 2010; 26: 2460–2461. 20709691 10.1093/bioinformatics/btq461

[42] Chia PL , Parakh S , Russell P *et al*. Expression of EGFR and conformational forms of EGFR in malignant pleural mesothelioma and its impact on survival. Lung Cancer 2021; 153: 35–41. 33453471 10.1016/j.lungcan.2020.12.028

