# Supplementary material for: Human EGFRvIII chimeric antigen receptor T cells demonstrate favorable safety profile and curative responses in orthotopic glioblastoma
Source: Clin Transl Immunology. 2023 Mar 5;12(3):e1440. doi: 10.1002/cti2.1440 (PMC9986233; doi:10.1002/cti2.1440)
Supplement: Supplementary file 1 — Supporting information [file CTI2-12-e1440-s001.docx]

**SUPPORTING INFORMATION**


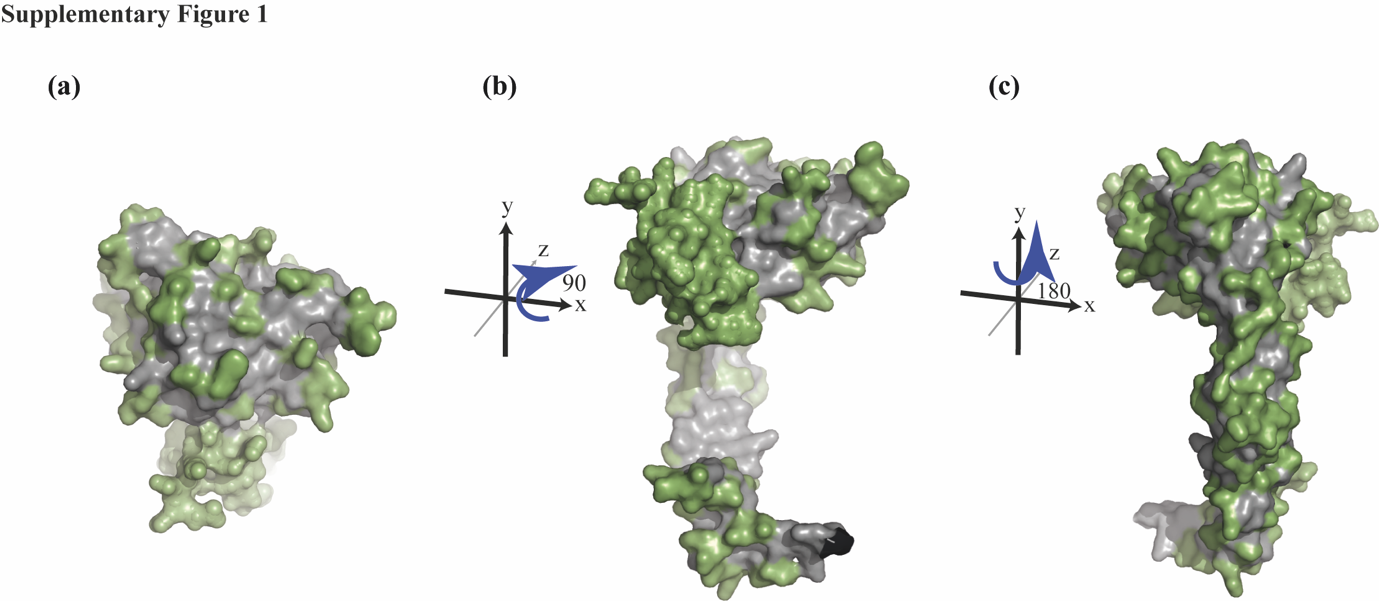


**Supplementary figure 1. Amino acid residues mutated to determine GCT02 predicted binding epitope.** Amino acid residues at positions 1–43 and the solvent exposed amino acids (positions 44–348) of the EGFRvIII extracellular domain (green) were mutated in the Deep Mutational Scanning approach to predict the GCT02 binding domain. Shown is the Alphafold structure of **(a)** the extracellular head **(b)** frontal view **(c)** backwards view of the EGFRvIII protein.


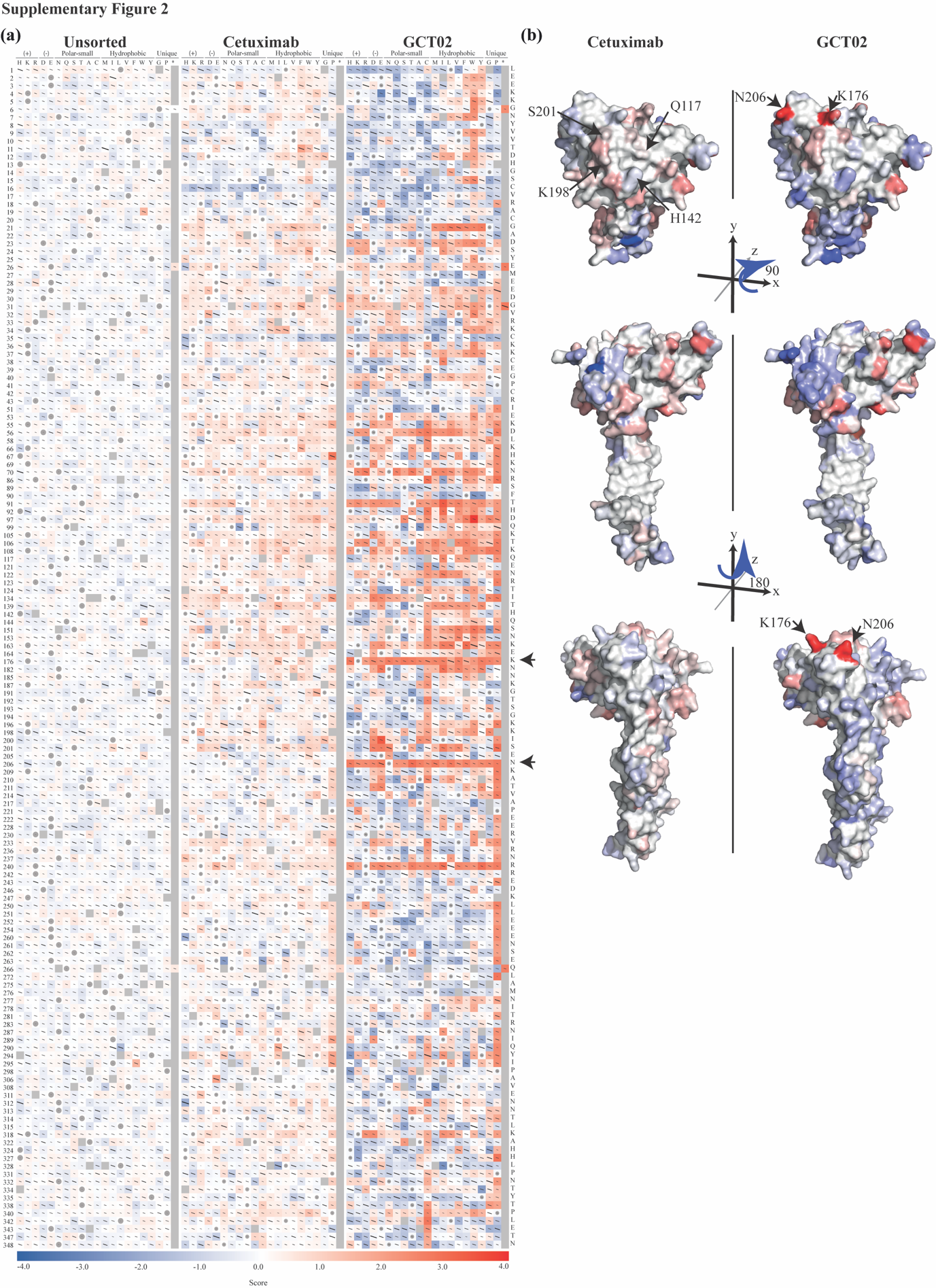


**Supplementary figure 2. Full sequence-function map generated to predict the binding epitope of GCT02.** The BW5147 cell line was transduced with the epitope mapping library, labelled with either Cetuximab or GCT02 and sorted for binding or non-binding. **(a)** Sequence-Function Maps of unsorted cells compared by Plasmid DNA, Cetuximab stained cells (non-binding versus binding) and GCT02 stained (non-binding versus binding) cells. Variant frequency in each cell population was used to calculate Log-Ratio scores that are used to colour the sequence-function maps. A score of 1 indicates and approximate 10-fold enrichment of that variant in the non-binding population. Positive enrichment scores are coloured red (non-binding variants), while negative enrichment scores are coloured blue (binding variants). Lines within each square represent standard error bars, with smaller bars indicating higher confidence. Squares containing a circle show the wild-type sequence. Grey squares denote no data. Alphafold model of EGFRvIII protein coloured by binding scores of **(b)** Cetuximab and **(c)** GCT02. The Log Ratio enrichment scores of Alanine, Serine, Threonine, Asparagine, Glutamine, Aspartic Acid, Glutamic Acid, Lysine, Arginine and Histidine variants were aggregated by position. The sum of Log Ratio scores was then used to replace the Cα B-factor of each position in an AlphaFold model of EGFRvIII. Residues that were not targeted in the DMS screen were set to 0. The surface was coloured by B-factor on a blue-white-red spectrum and scaled such that blue and red extended equally into the negative and positive scale and set to the maximise the contrast of each dataset: Cetuximab ± 12.69 and GCT02 ± 22.81. Red indicates variants that are enriched in the non-binding population. Blue indicates variants that are enriched in the binding population.

.


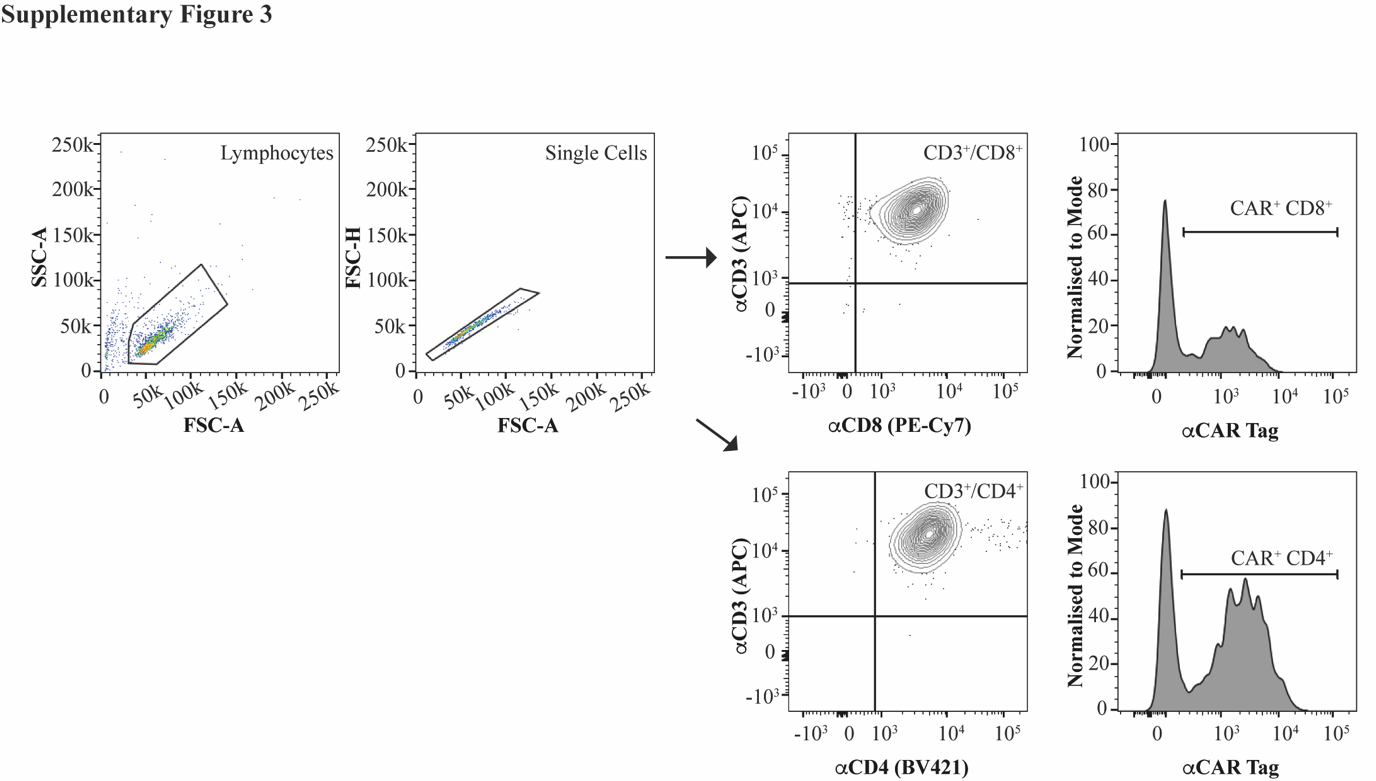


**Supplementary figure 3. Flow cytometry gating strategy to determine the expression of the GCT02 or 2173 CAR on primary human T cells.** Flow cytometry gating strategy to identify primary human CAR T cells. Cells expressing CD3^+^/CD4^+^ or CD3^+^/CD8^+^ were defined as T cells, and CAR expressing cells were defined as those expressing MYC-tag (GCT02) or FLAG-tag (2173). Data are representative of six experiments with four individual donors.


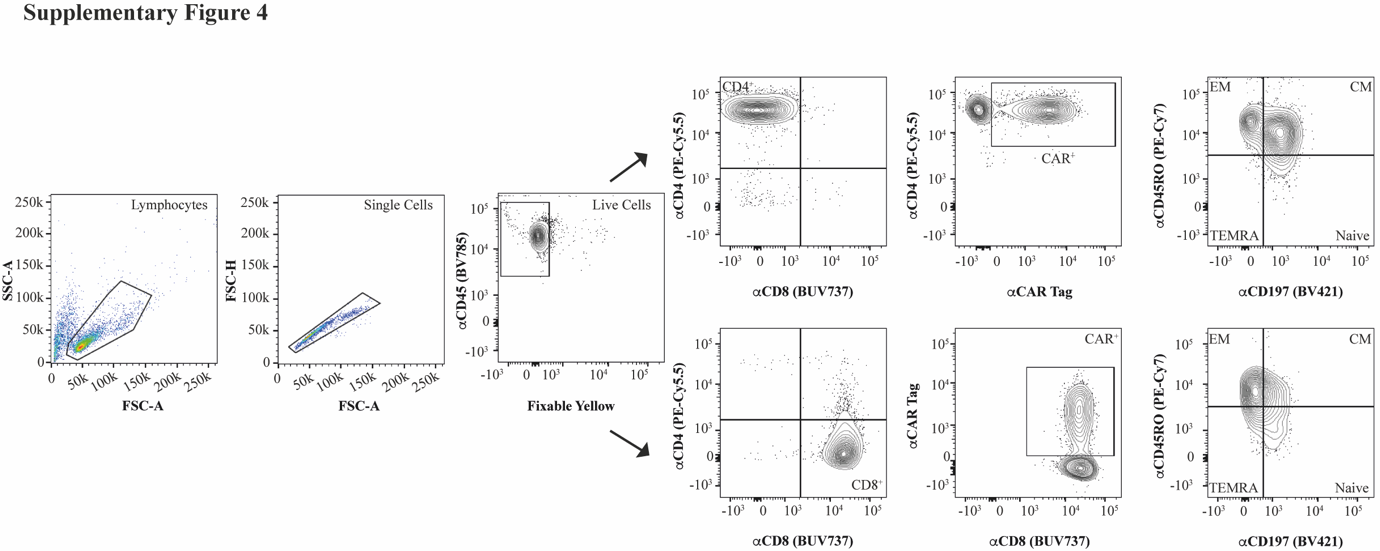


**Supplementary figure 4. Flow cytometry gating strategy to determine the phenotype of the GCT02 or 2173 human CAR T cells.** Flow cytometry gating strategy to determine the phenotypic profile of primary human CAR T cells. CAR T cells were defined as live, CD45^+^ and either CD4^+^ or CD8^+^, expressing the αMYC-tag (GCT02) or FLAG-tag (2173). The figure demonstrates the gating strategy using GCT02 transduced T cells but applies to both treatment groups. T cell phenotype was determined based on expression of CD45RO and CD197. Data are representative of two experiments with two individual donors.


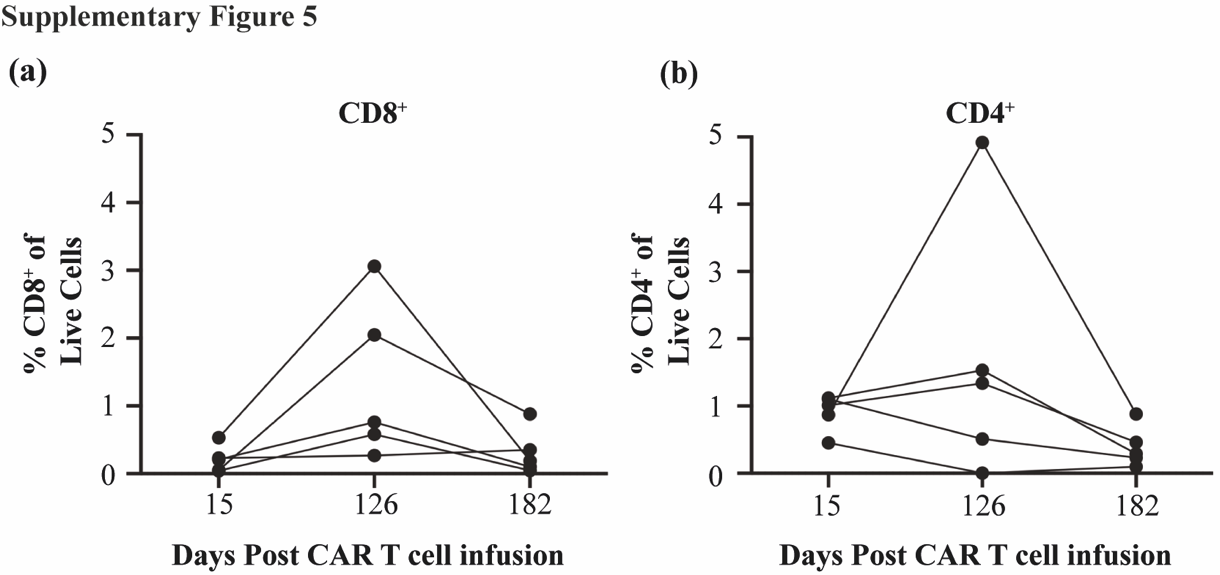


**Supplementary figure 5. GCT02 CAR T cells persist in treated U87-EGFRvIII tumour-bearing mice.**

Peripheral blood samples were collected from mice bearing U87-EGFRvIII tumours, treated with GCT02 CAR T cells at days 15, 126 and 182. The samples were analysed by flow cytometry to identify persisting **(a)** CD8^+^ and **(b)** CD4^+^ T cells. Each line represents the percentage of T cells identified in a single mouse. The experiment was performed once, N = 5 mice.


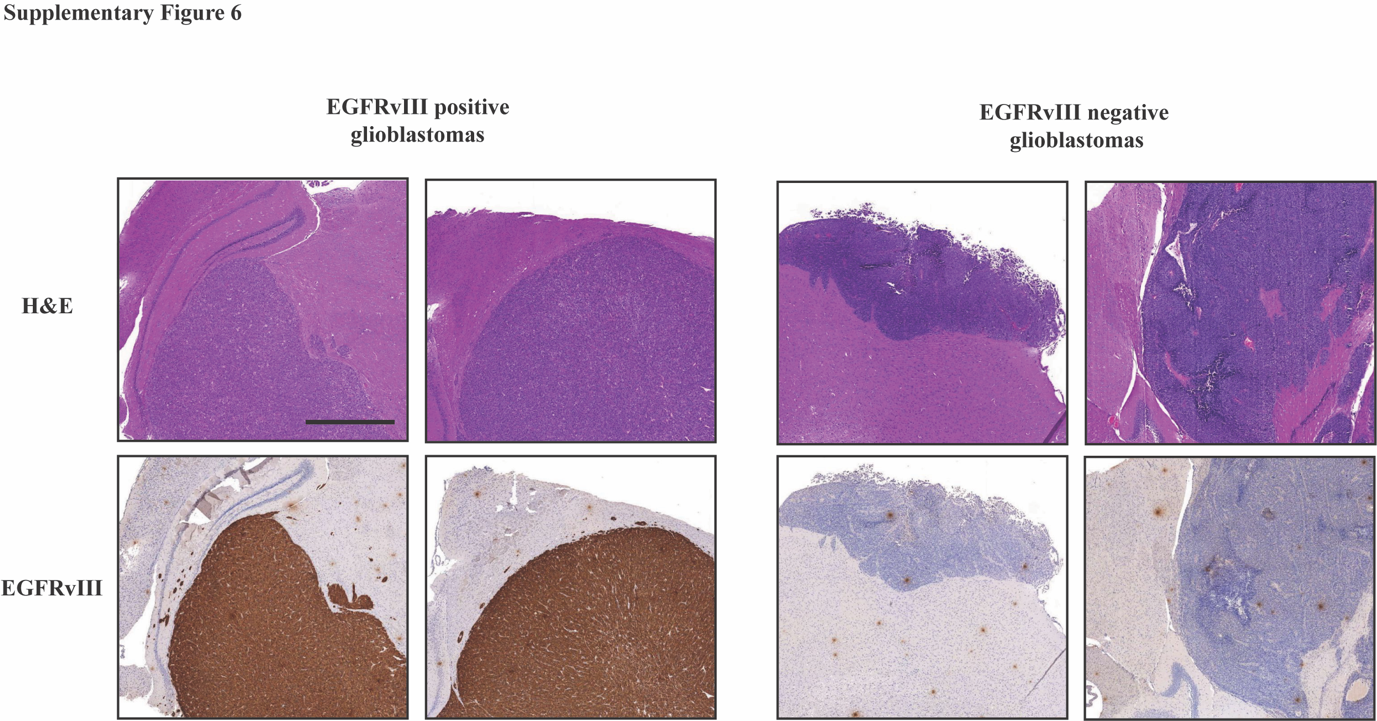


**Supplementary figure 6. EGFRvIII antibody specifically binds to EGFRvIII tumour samples.**

Brain sections were prepared from untreated, U87-EGFRvIII (left) or U87 tumour-bearing mice (right). The brain sections were stained with H&E (top row) or the LMH-151 EGFRvIII antibody. Shown are brain sections from two mice with an EGFRvIII-expressing tumour and two mice with an EGFRvIII-negative tumour at 2X magnification. The scale bar is 1000 μM and applies to all images.


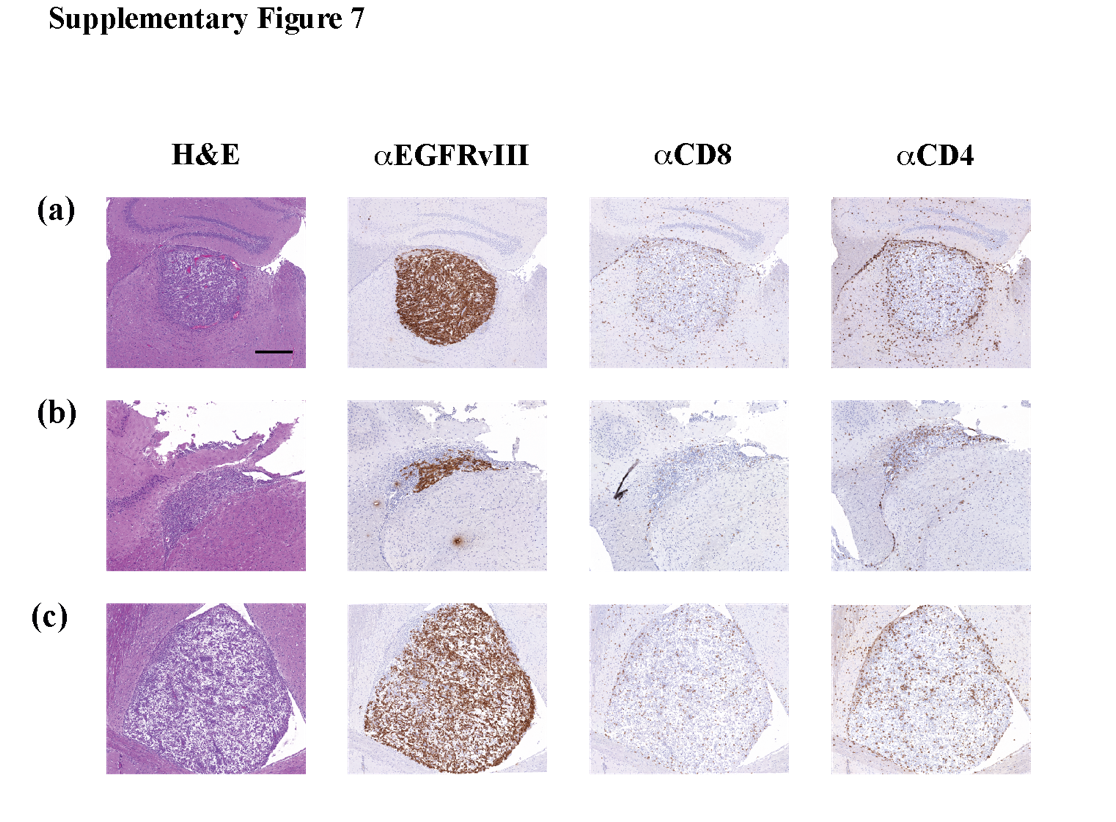


**Supplementary figure 7. Analysis of EGFRvIII expression and infiltrating GCT02 CAR T cells in U87-EGFRvIII intracranial tumour-bearing mice.** One week post CAR T cell infusion, tumour-bearing mice were euthanised and the fixed brains were analysed by immunohistochemistry. **(a–c)** Brain sections from three individual mice, which are labelled with haematoxylin and eosin and antibodies to EGFRvIII, CD8 and CD4. The scale bar is 500 μM and applies to all images. The images depicted in **a** are also shown in **Figure 5h–k**.


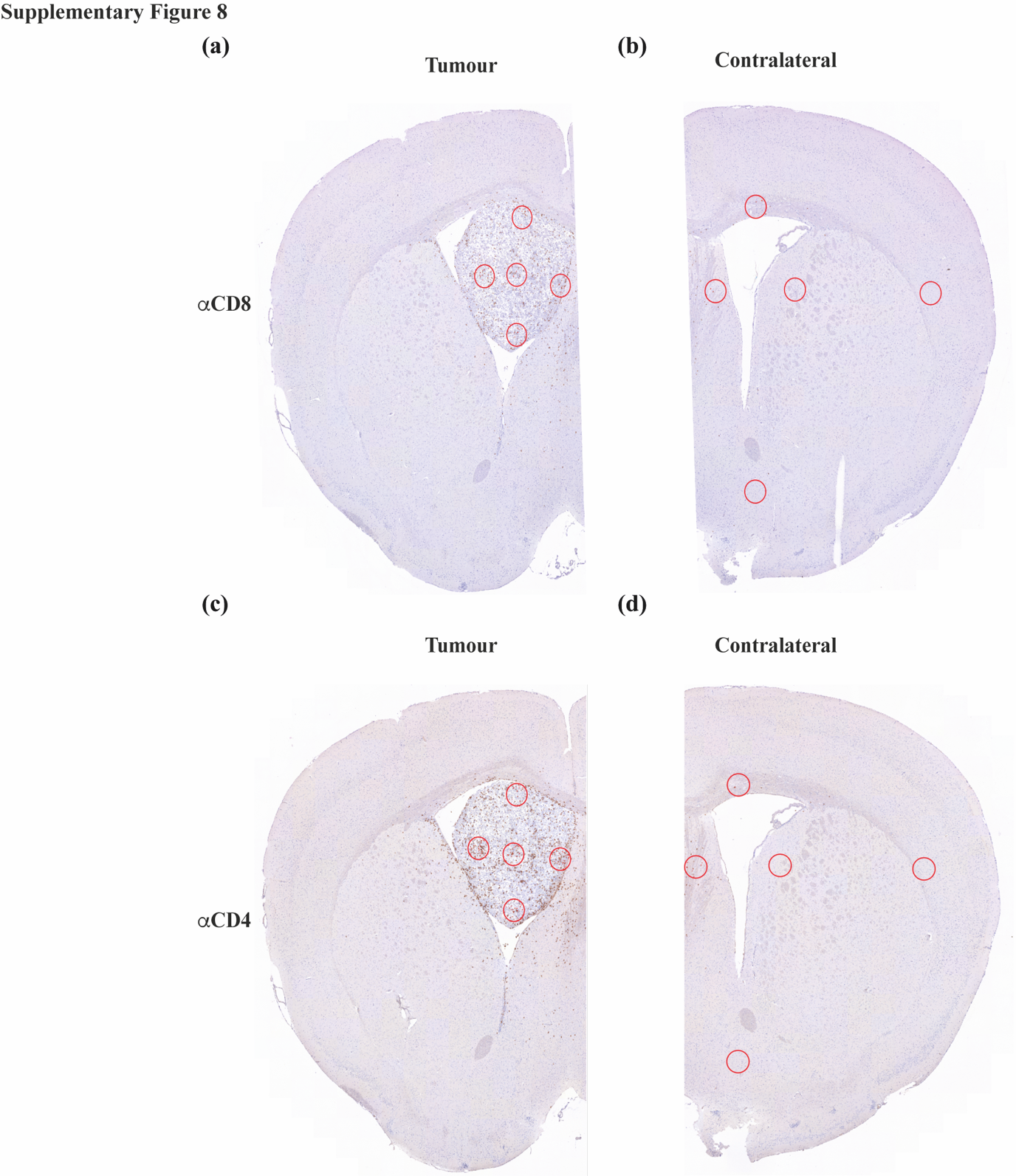


**Supplementary figure 8. Regions of quantification of infiltrating T cells in tumour-bearing mice.** One week post CAR T cell infusion, tumour-bearing mice were euthanised and the fixed brains were analysed by immunohistochemistry. CD4^+^ and CD8^+^ T cell infiltration was quantified in three independent mice in five regions (red circles) from both the tumour-bearing **(a), (c)** or contralateral **(b)**, **(d)** hemispheres. Shown is a representation of five randomly selected regions (red circles) in both the tumour-bearing and contralateral brain hemispheres.


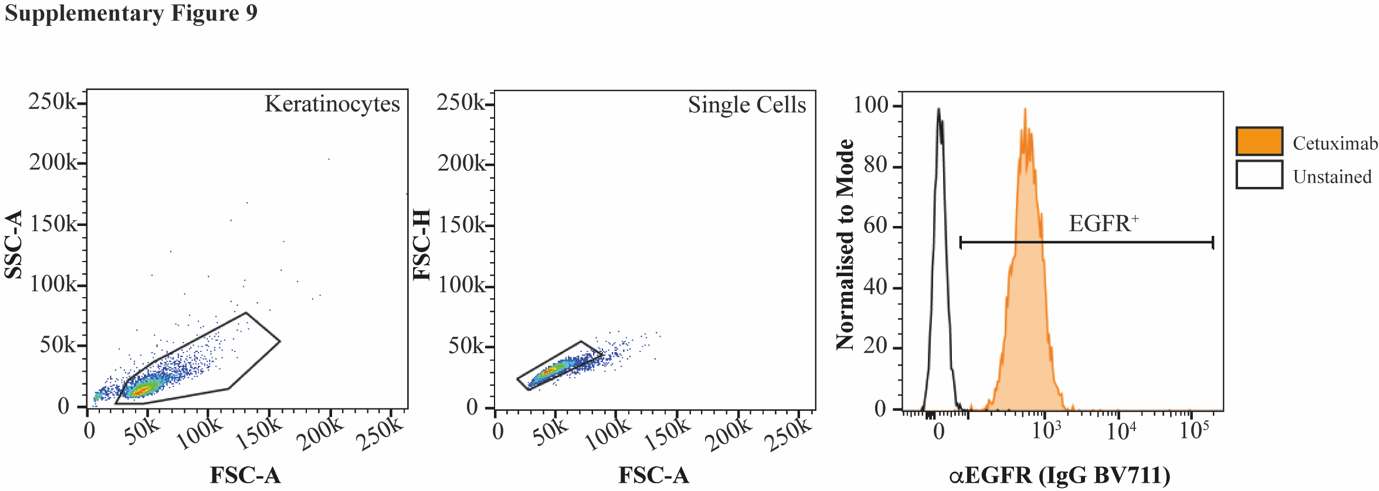


**Supplementary figure 9. Flow cytometry gating strategy to determine the expression of EGFR on primary human keratinocytes.** Flow cytometry gating strategy to determine the expression of EGFR on primary human keratinocytes via cell surface labelling with Cetuximab. Data are representative of one experiment with three individual donors.


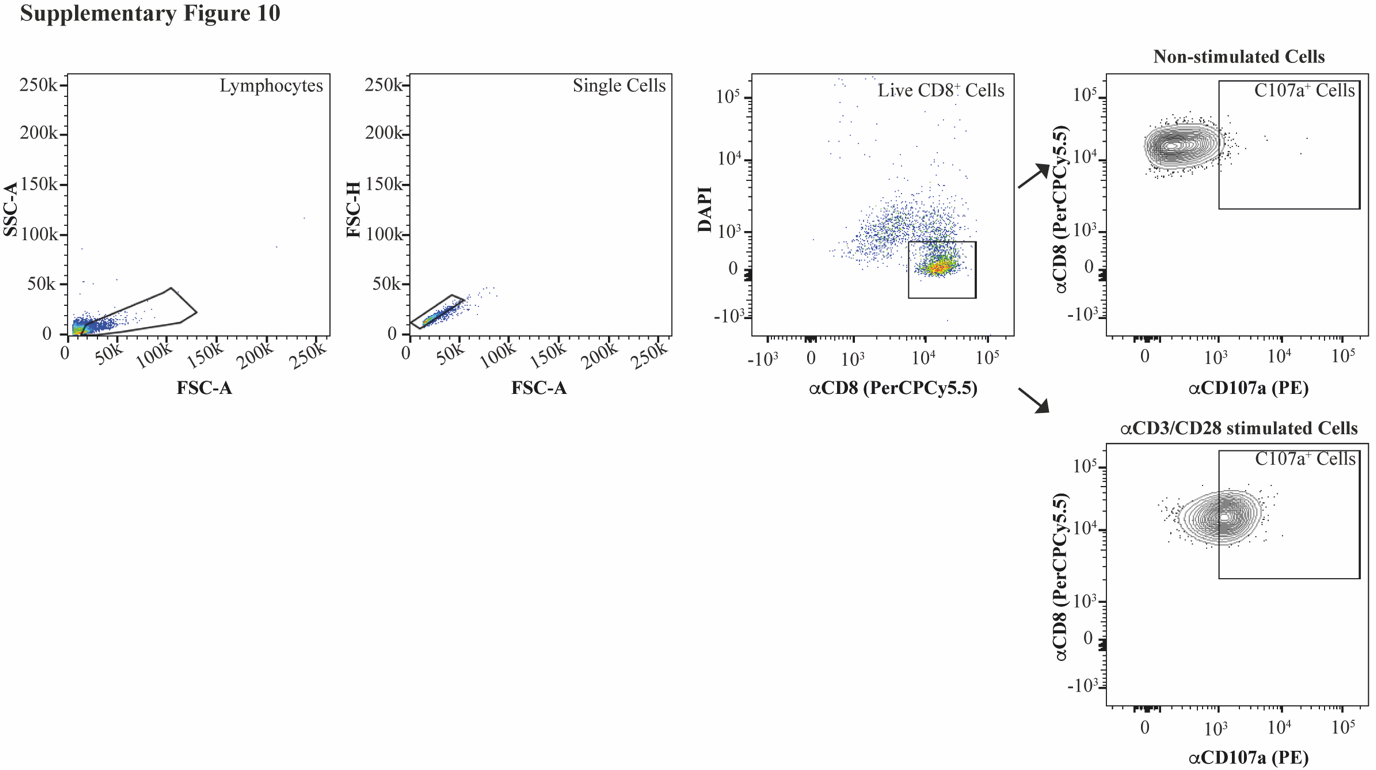


**Supplementary figure 10: Flow cytometry gating strategy to determine T cell degranulation.** Flow cytometry gating strategy to determine human T cell degranulation post coculture with different stimuli. Degranulation was determined as CD8^+^ T cells positive for CD107a. Data are representative of one experiment, with three individual T cell donors each assayed in triplicate.

**Supplementary table 1.** List of flow cytometry antibodies

| **TARGET ANTIGEN** | **FLUOROPHORE CONJUGATE** | **CLONE** | **ISOTYPE** | **MANUFACTURER** |
| --- | --- | --- | --- | --- |
| FLAG-tag | FITC | M2 | IgG1 | Sigma Aldrich, St Louis, USA |
| MYCtag | AF647, AF488 | 9B11 | IgG2a | Sigma Aldrich, St Louis, USA |
| CD107a | PE | H4A3 | IgG1 | Becton Dickenson Biosciences, New Jersey, USA |
| CD197 | BV786 | 3D12 | IgG2a | Becton Dickenson Biosciences, New Jersey, USA |
| CD3 | APC | SK7 | IgG1 | Becton Dickenson Biosciences, New Jersey, USA |
| CD4 | BV421 | OKT4 | IgG2b | Biolegend, San Diego, USA |
| CD4 | PECy5.5 | S3.5 | IgG2a | Invitrogen, Waltham, USA |
| CD45 | BV785 | HI30 | IgG1 | Biolegend, San Diego, USA |
| CD45RO | PE-Cy7 | UHCL1 | IgG2a | Becton Dickenson Biosciences, New Jersey, USA |
| CD8 | PECy7 | RPA-T8 | IgG1 | Becton Dickenson Biosciences, New Jersey, USA |
| CD8 | BUV737 | SK1 | IgG1 | Becton Dickenson Biosciences, New Jersey, USA |
| EGFR (Cetuximab) | Unconjugated | NA | IgG1 | Merck Serono, Darmstadt, Germany |
| EGFRvIII | Unconjugated | GCT02 | IgG | ATUM, Newark, USA |
| mouse anti-human | BV711 | NA | IgG | Becton Dickenson Biosciences, New Jersey, USA |

**Supplementary table 2.** List of histology antibodies

| **TARGET** | **ISOTYPE** | **MANUFACTURER** |
| --- | --- | --- |
| Anti-Rabbit-HRP | IgG | Nichirei Biosciences Inc  Chūō, Japan |
| Anti-Rat-HRP IgG | IgG | Vector Laboratories, Newark, USA |
| CD4 | IgG | Abcam, Cambridge, UK |
| CD8 | IgG | Invitrogen, Waltham, USA |
| EGFRvIII  (Clone LMH-151) | IgG3 | A kind gift from Professor Andrew Scott Olivia Newton John Cancer Research Institute, Heidelberg, Australia |
